# Enhancing the immunogenicity of Wilms tumor 1 epitope in mesothelioma cells with immunoproteasome inhibitors

**DOI:** 10.1371/journal.pone.0308330

**Published:** 2024-08-08

**Authors:** Masaki Ito, Shigeo Koido, Takeo Iwamoto, Soyoko Morimoto, Fumihiro Fujiki, Haruo Sugiyama, Saki Matsumoto, Clara Effenberger, Kazuma Kiyotani, Kiyotaka Shiba

**Affiliations:** 1 Institute of Clinical Medicine and Research, Research Center for Medical Sciences, The Jikei University School of Medicine, Chiba, Japan; 2 Cancer Institute, Japanese Foundation for Cancer Research, Tokyo, Japan; 3 The Division of Gastroenterology and Hepatology, Department of Internal Medicine, The Jikei University School of Medicine, Kashiwa Hospital, Chiba, Japan; 4 Core Research Facilities, The Jikei University School of Medicine, Tokyo, Japan; 5 Department of Cancer Stem Cell Biology, Osaka University Graduate School of Medicine, Osaka, Japan; 6 Department of Cancer Immunology, Osaka University Graduate School of Medicine, Osaka, Japan; 7 Project for Immunogenomics, Cancer Precision Medicine Center, Japanese Foundation for Cancer Research, Tokyo, Japan; Baylor College of Medicine, UNITED STATES OF AMERICA

## Abstract

The immunogenicity of cancer cells is influenced by several factors, including the expression of the major histocompatibility complex class I (MHC-I), antigen expression, and the repertoire of proteasome-produced epitope peptides. The malignant pleural mesothelioma cell line ACC-MEOS-4 (MESO-4) expresses high levels of MHC-I and Wilms tumor 1 (WT1) tumor antigens. Using a functional T cell reporter assay specific for the HLA-A*24:02 restricted WT1 epitope (WT1_235_, CMTWNQMNL), we searched for factors that augmented the immunogenicity of MESO-4, focusing on proteasomes, which have a central role in the antigen processing machinery. ONX-0914, a selective inhibitor of the immunoproteasome subunit β5i, enhanced immunogenicity dose-dependently at low concentrations without cytotoxicity. In addition, CD8^+^ T lymphocytes recognizing WT1 showed greater cytotoxicity against MESO-4 pre-treated with ONX-0914. MESO-4 expresses a standard proteasome (SP) and immunoproteasome (IP). Notably, IP has distinct catalytic activity from SP, favoring the generation of antigenic peptides with high affinity for MHC-I in antigen-presenting cells and cancer cells. *In vitro*, immunoproteasome digestion assay and mass spectrometry analysis showed that IP cleaved WT1_235_ internally after the hydrophobic residues. Importantly, this internal cleavage of the WT1_235_ epitope was mitigated by ONX-0914. These results suggest that ONX-0914 prevents the internal destructive cleavage of WT1_235_ by IP, thereby promoting the specific presentation of the WT1 epitope by MESO-4. In conclusion, selective IP inhibitors might offer a means to modulate cancer cell immunogenicity by directing the presentation of particular tumor epitopes.

## Introduction

Approximately 25,000 people died from mesothelioma worldwide in 2018 (1) and 1605 in Japan in 2020 [1]. Malignant pleural mesothelioma (MPM) is the most common form of mesothelioma and a progressive and refractory cancer. MPM develops from squamous cells of mesodermal origin that form the inner layer of the pleura, mostly due to long-term occupational exposure to asbestos. MPM has a poor prognosis and is resistant to standard chemotherapy with cisplatin (platinum) and pemetrexed (folate metabolism antagonist). Recently, immunotherapy with nivolumab (anti-PD1 antibody) and ipilimumab (anti-CTLA-4 antibody) was approved in Japan and the USA as the first-line treatment for unresectable MPM [2]. However, MPM is still highly resistant to current therapeutic modalities. Therefore, the development of innovative therapies for MPM is required [3].

Wilms Tumor 1 (WT1) is a zinc finger transcription factor widely expressed in mesoderm-derived tissues during embryonic development, including mesothelial tissue, kidney, gonads, heart, and spleen. In adult tissues, WT1 expression is restricted to mesothelium, hematopoietic stem cells, myoepithelial progenitor cells, renal sheath cells, and some cells in the testis and ovary [4–6]. WT1 is also involved in the carcinogenic process and is overexpressed in MPM, leukemia, and various solid tumors [7]. Because of the specific expression of nuclear WT1 in mesothelioma, immunohistochemical staining for WT1 is used as a diagnostic marker to distinguish mesothelioma from lung cancer [8]. Clinical trials of acute myeloid leukemia (AML), advanced pancreatic cancer, and MPM have been conducted with the WT1 peptide vaccine [9–12]. In addition, engineered T cells transfected with the WT1-specific T cell receptor (TCR) gene were developed as an adaptive T cell therapy [13, 14]. These reports suggest that induction of immunity against the endogenous WT1 antigen provides anti-tumor immunity, even though WT1 is not a neo-antigen with highly immunogenic amino acid substitutions caused by genetic mutations.

The immunogenicity of cancer cells is defined by the presentation of antigenic epitopes on MHC-I. Mechanisms that reduce antigen presentation on the tumor cell surface include decreased MHC-I expression, decreased antigen expression, and altered MHC-I epitope repertoire due to abnormalities in components of the antigen processing and presentation machinery [15]. Most antigenic epitope peptides are generated by the degradation of endogenous proteins by the proteasome, a multi-subunit enzyme complex [16, 17].

There are several types of proteasomes, including standard proteasomes (SP), immunoproteasomes (IP), and intermediate proteasomes (IMP), which are hybrid proteasomes of SP and IP [15, 16, 18, 19]. IP is expressed in tumor cells under inflammatory conditions in response to interferon-gamma (IFNγ) but is constitutively expressed in specialized antigen-presenting cells such as dendritic cells. The IP replaces the three catalytic subunits of SP (β1c, β2c, and β5c) with IFNγ-induced alternative catalytic units (LMP2/β1i, MECL-1/β2i, and LMP7/β5i) with different cleavage specificities [15, 20, 21]. IP overexpression was reported in various cancers, including AML, breast cancer, and melanoma [18, 19]. In melanoma, β5i and β1i are biomarkers for immune checkpoint inhibitor therapy because they alter the immunopeptidome and induce a higher level of immune response [17]. The IP may destroy some antigenic peptides, such as tyrosinase_369_ (YMDGTMSQV), due to its higher chymotrypsin-like activity, rather than favoring their production [22]. IP triplex-deficient mice (β1i, β2i, β5i) are viable, but their immunopeptidome was reported to differ by ~50% from that of wild mice [23].

Antigen presentation of the HLA-A*02-restricted WT1_126_ epitope (WT1_126_, RMFPNAPYL), which is immunoproteasome β1i-dependent, was reduced by immunotherapy-induced downregulation of the immunoproteasome in acute myeloid leukemia cells [21]. Moreover, the WT1_126_ epitope is not a suitable target for T cell-based immunotherapy due to its low presentation on MHC-I, despite the high expression of WT1 antigen in leukemia cells [20].

Thus, cellular immunogenicity is influenced by the expressions of MHC-I and target antigens, and by epitope processing by the proteasome. Therefore, controlling proteasome functions might influence the repertoire of epitope peptides presented to cytotoxic T-cells (CTLs) to induce stronger immune responses against cancer cells.

We report that ONX-0914, a selective inhibitor of the immunoproteasome LMP7/β5i, enhanced the immunogenicity of malignant pleural mesothelioma cells against WT1-specific T cells. Our findings will aid the development of complementary therapies combining WT1 epitope-specific immunotherapy and IP-selective inhibitors.

## Materials and methods

### Cell lines

MPM, ACC-MESO-4 (MESO-4), ACC-MESO-1 (MESO-1), and HMMME cell lines were provided by RIKEN BRC through the National BioResource Project of the MEXT/AMED, Japan [24]. The T2-A24 cell line was kindly provided by Kazushima K. (Division of Virology, Aichi Cancer Center Research Institute, Japan). MESO-1, MESO-4, and T2-A24 cell lines were cultured in RPMI 1640, L-glutamine (Nacalai Tesque, #30264–56) supplemented with 10% FBS (Corning, #35-079-CV). The HMMME cell line was cultured in Ham’s F-12, L-glutamine, sodium pyruvate (Nacalai Tesque, #17458–65) supplemented with 15% FBS. TrypLE Express (Thermo Fisher Scientific, #12605010) was used to dissociate tumor cells from culture dishes. B10-TCR td 2D3 cells (reporter T cells) that specifically react to the WT1 epitope presented on HLA-A*24:02 (WT1_235_, CMTWNQMNL) or a modified WT1 epitope (WT1_M236Y_, CYTWNQMNL) were developed previously [25]. Reporter T cells were cultured in RPMI 1640 supplemented with 50 μM 2-mercaptoethanol (Sigma), 1% nonessential amino acids (Nacalai Tesque, #06344–56), and 10% FBS. All cell lines were verified free of mycoplasma (TaKaRa, PCR Mycoplasma Detection Set, #6601).

### *In vitro* functional immunogenicity assay

The functional T-cell-based reporter assay using the B10-TCR td 2D3 cell line (reporter T cells) is used to evaluate the effect of drugs on tumor cell immunogenicity [25]. Reporter T cells were engineered to express a WT1_235_-specific TCR (B10-TCR) and a reporter construct containing a nuclear factor of activated T cells (NFAT) transcription factor response element and an IL-2 minimal promoter. When the TCR recognizes the MHC-I/WT1 peptide complex on target tumor cells, activated TCR signaling induces green fluorescent protein (GFP) expression. Target tumor cells were seeded in 96-well flat-bottomed polystyrene plates, 5x10^3^ or 1x10^4^ cells/well, and incubated overnight in 2:1:1 medium (RPMI:DMEM:Ham’s F-12). Tumor cells were cocultured with varying ratios of reporter T cells. After 20 hours of coculture, reporter T cells were stained with anti-CD8 antibody (BioLegend, clone RPA-T8, #301014), and tumor cell immunogenicity was determined by detecting GFP-positive reporter T cell events (GFP^+^) by flow cytometry (Miltenyi Biotech, MACSQuant Analyzer 10). The gating strategy for GFP^+^-activated T cells is shown in S1 Fig [26].

### Inhibitors and chemicals

Bortezomib (#PS-341) was from Funakoshi, MG132 (#3175-v) and epoxomicin (#4381-v) were from Nacalai Tesque, and ONX-0914 (#16271) was from Cayman Chemical. PMA (#P8139) and Ionomycin (#I9667) were from Sigma. IFNγ (#300–02) was from PeproTech.

### Flow cytometric analysis

Flow cytometric analysis was performed using a MACSQuant Analyzer 10 (Miltenyi Biotech). To investigate MHC-I expression in MPM cell lines, cells were stained with anti-β2-microglobulin-associated MHC-I complexes (HLA-A, -B and -C alleles) (BioLegend, clone W6/32, #311404) and anti-HLA-A*24 (MBL, clone 22E1, #K0209-5).

### Reverse Transcription-Polymerase-Chain Reaction (RT-PCR) analysis

Total RNA was prepared from mesothelioma cells using the QIAGEN RNeasy Plus Mini Kit (QIAGEN, #74134). RNA (2 μg) was subjected to multiplex RT-PCR using the OneStep RT-PCR Kit (QIAGEN, #210210) according to the manufacturer’s instructions. Reverse transcription and amplification were performed using the following parameters: 50°C for 30 minutes; 95°C for 15 minutes, then 94°C for 1 minute, 60°C for 1 minute, and 72°C for 2 minutes, repeated for 30 cycles; 72°C for 10 minutes, followed by a 4°C hold. RT-PCR products were electrophoresed on 2% agarose gels (Lonza, MetaPhor^™^ agarose) containing ethidium bromide for 45 minutes at 100 V, and then detected under UV transillumination. Primers were: LMP7 (F 5’-gaacacttatgcctacggggtc-3’, R 5’-tttctactttcacccaaccatc-3’), LMP2 (F 5’-gggatagaactggaggaacc-3’, R 5’-agatgacacccccgcttgag-3’), and GAPDH (F 5’-aggggggagccaaaaggg-3’, R 5’-gaggagtgggtgtcgctgttg-3’) [27].

### Lactate dehydrogenase (LDH) cytotoxicity assay

WT1-reactive CD8-positive T cells (WT1-CTLs) were induced using a modified WT1_M236Y_ epitope peptide from human peripheral blood mononuclear cells (PBMCs) from pancreatic cancer patients vaccinated with WT1_M236Y_ pulsed dendritic cells [28, 29]. MESO-4 were pre-cultured with ONX-0914 for 18 hours and cocultured with WT1-CTLs in 1% human AB serum/RPMI without phenol red medium (Nacalai Tesque, #06261) for 4 hours. The cytotoxicity of WT1-CTLs was assessed using the cytosolic enzyme LDH cytotoxicity assay kit (Nacalai Tesque, #18250).

### Proteasome digestion assay

The proteasome digestion assay was performed as previously described [30]. The precursor peptide containing the WT1_235_ epitope (TSQLE**CMTWNQMNL**GATLK) was synthesized by GenScript and was >95% pure. 20S SP from human erythrocytes (#SBB-PP0005) and 20S IP from human PBMCs (#SBB-PP0004) were purchased from South Bay Bio (San Jose, CA, USA). Precursor peptides (10 μg/300 μl) were incubated with 0.5 μg of SP or IP in reaction buffer (20 mM HEPES-KOH pH7.8, 2 mM MGAc2, 2 mM dithiothreitol) at 37°C for 3 hours. Samples were processed for LC-MS/MS and analyzed as previously described [31].

### Western blot analysis

Tumor cells were incubated with 20 nM ONX-0914 and 10 ng/mL IFNγ (Sigma-Aldrich, #L2880) for 3 hours, washed with PBS, and lysed with RIPA buffer containing proteinase inhibitor (Nacalai Tesque, #08714–04). Total cell extracts were resolved on 4–12% SDS-PAGE gels and analyzed by western blotting using antibodies against WT1 (clone D8I7F, #83535), β1c (PSMB6, clone E1K9O, #13267), β2c (PSMB7, clone E1LSH, #12197), β2i (MECL-1/PSMB10, clone E6R7O, #17579), β5c (PSMB5, clone D1H6B, #12919), and β5i (LMP-7/PSMB8, clone D1K7X, #13635) (Cell Signaling Technology), β1i (Santa Cruz Biotechnology, PSMB9/LMP2, clone G-3, #373996), and GAPDH as a loading control (BioLegend, #649204).

### Ethics statement

This study was performed according to guidelines approved by The Ethics Committee of the Jikei University School of Medicine for Biomedical Research (#30–303 and #21–204).

Peripheral blood mononuclear cells stored in a previously conducted clinical trial (#21–204) were used in this study on 5 April 2023. We did not access any information that could identify individuals who participated in #21–204 [28].

### Statistical analysis

Statistical significance was determined by unpaired t-test (for comparisons between two groups, two-tailed), one-way ANOVA (for comparisons of three or more groups), and Tukey’s multiple comparison test and Dunnett’s multiple comparison tests where appropriate using GraphPad Prism 9.4.0 software (GraphPad).

## Results

### Reporter T cells detect WT1-specific immunogenicity of cancer cells

**Fig 1:**
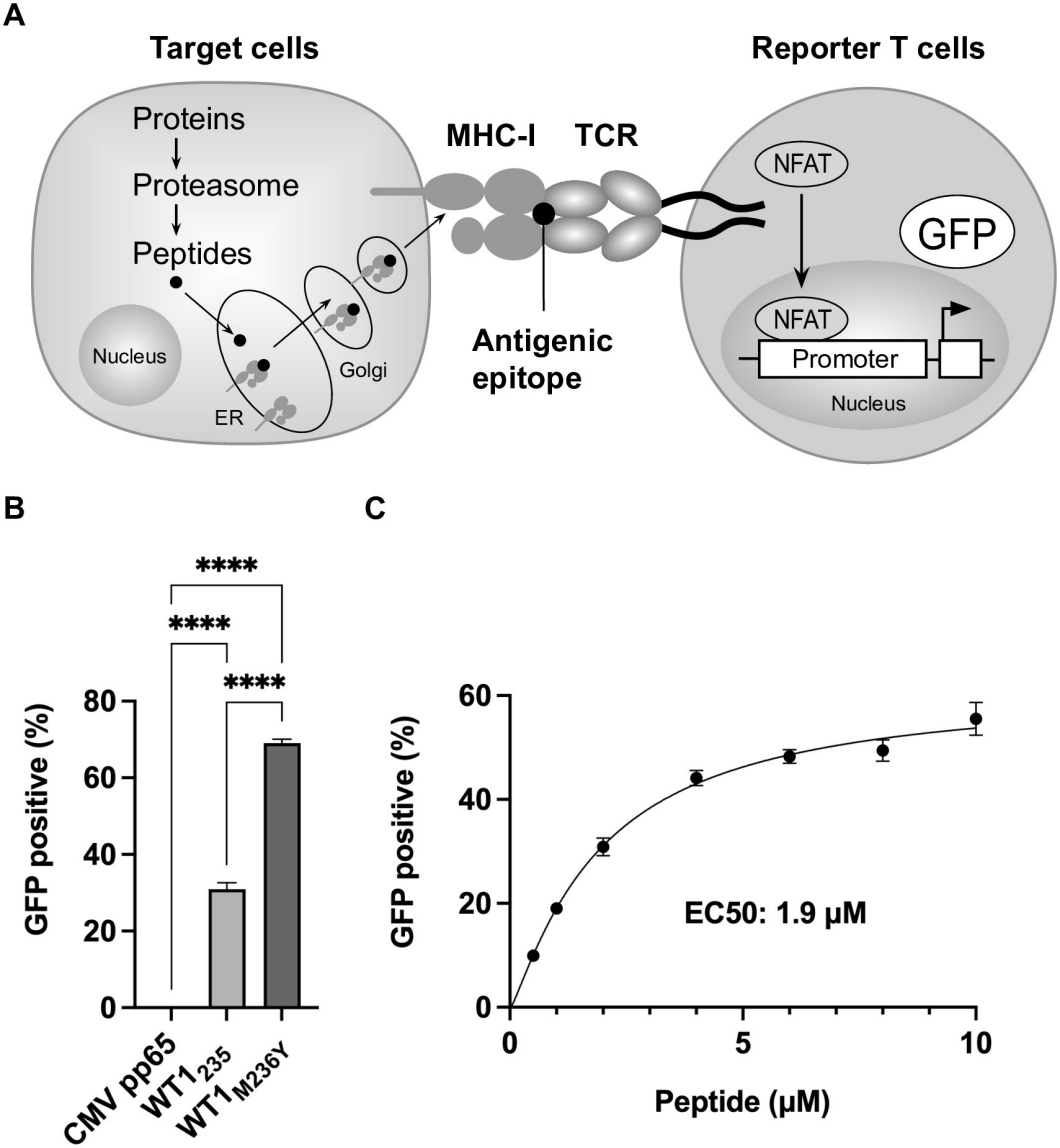
Cell immunogenicity monitoring using antigen-specific reporter T cells. (**A**) Schematic representation. Reporter T cells (B10-TCR td 2D3 cell line) express WT1_235_ peptide (CMTWNQMNL)-specific TCR and a TCR signaling reporter construct. The reporter construct comprises 3x nuclear factor of activated T cells (NFAT)-response elements, IL-2 minimal promoter, and GFP. When the target cells and reporter T cells are cocultured, the interaction between WT1-specific TCR and WT1_235_ peptide/MHC-I complex activates TCR signaling. Upstreaming signaling events result in NFAT nuclear translocation and subsequent response-element binding. The response element, in turn, drives the expression of GFP. (**B**) Specificity of reporter T cells. T2-A24 cells were pulsed with 10 μM of the indicated HLA-A24-restricted peptides. WT1_M236Y_ is a single amino acid substitution (M→Y) at position 2 of the native 9-mer WT1_235_, improving peptide/HLA-A*24 MHC-I complex stability. Cellular immunogenicity was evaluated by monitoring the activated reporter T cells with GFP expression (GFP positive %) using flow cytometry (S1 Fig). Data are represented as the mean ± SD of three independent experiments and Tukey’s multiple comparisons test was used. ****, p<0.0001. (**C**) Quantification of natural WT1_235_ epitopes on T2-A24 cells using reporter T cells.

To assess the immunogenicity of cancer cells, we used a functional reporter system that expresses GFP upon antigen-specific response (Fig 1A). The protein is processed into short peptides by the proteasome in the cytoplasm, translocated to the endoplasmic reticulum lumen, bound to MHC-I molecules, and presented on the cell surface. When reporter T cells recognize epitopes presented on the MHC-I of target cells with antigen-specific TCR, TCR signaling is activated, NFAT transcription factors translocate to the nucleus, and GFP expression is induced. The antigen-specific immunogenicity of target cells can be assessed by measuring the number of GFP-positive cells by flow cytometry.

First, we reconfirmed the antigen specificity of the reporter T cells previously generated [25]. T2-A24 cells lack the transporter for antigen presentation (TAP) and do not express the endogenous antigen epitope on MHC-I molecules. WT1_235_ peptide (CMTWNQMNL), WT1_M236Y_ peptide (CYTWNQMNL), and human cytomegalovirus pp65 peptide (CMV pp65_341_, QYDPVAALF) were pulsed on T2-A24 cells. WT1_M236Y_ has a single amino acid substitution (M→Y) at position 2 of the native 9-mer WT1_235_ peptide to improve the stability of the peptide/HLA-A*24 MHC-I complex. Reporter T cells were cocultured with peptide-pulsed T2-A24 cells and activated GFP-positive reporter T cells recognizing the WT1 epitope displayed on HLA-A*24 molecules were analyzed by flow cytometry (S1 Fig). Reporter T cells responded to WT1_235_ and WT1_M236Y_ peptides but not to CMV pp65_341_ (Fig 1B). WT1_M236Y_ stimulated reporter T cells at significantly higher levels than the original WT1_235_ (p<0.0001). Reporter T cells responded to the WT1_235_ peptide dose-dependently (Fig 1C). Thus, reporter T cells can quantitatively evaluate WT1 epitopes presented on HLA-A*24 molecules on target cells.

### MPM cell lines express high levels of WT1 antigen

**Fig 2:**
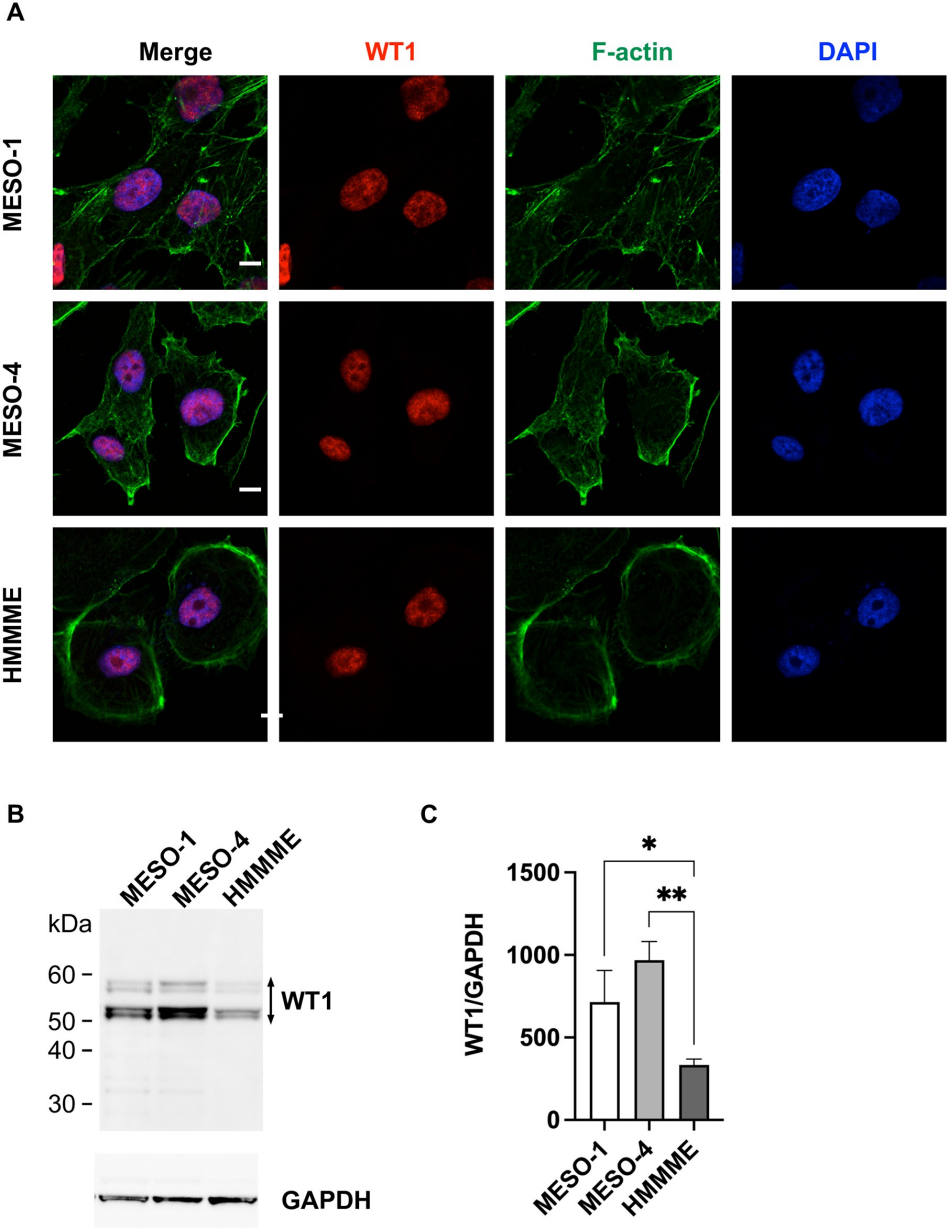
WT1 expression in mesothelioma cells. (**A**) Immunofluorescence staining of WT1. Mesothelioma cells were stained with anti-WT1 antibody (WT1) (red), F-actin was stained with Alexa Fluor 488 Phalloidin (green), and cell nuclei were stained with DAPI (blue). Scale bar = 10 μm. (**B**) Western blot of WT1 and GAPDH in mesothelioma cells. (**C**) Densitometry values of expression relative to GAPDH are indicated. Data are represented as the mean ± SD of three independent experiments and Tukey’s multiple comparisons tests. *, p<0.05; **, p<0.01.

WT1 is a reliable marker of mesothelial cell lineage and is highly expressed in normal adult lung mesothelium [7, 8]. We investigated the expression of WT1 in MPM cell lines. WT1 was exclusively localized to the nucleus in three MPM cell lines with epithelial morphology (MESO-1, MESO-4, HMMME), indicating WT1 is a transcription factor (Fig 2A). At least 20 different WT1 proteins are generated by alternative mRNA splicing [32]. Western blotting using anti-WT1 antibodies recognizing the amino terminus of WT1 detected four different sizes of WT1, which are thought to be modified WT1 (Fig 2B). Total WT1 protein expression was significantly high in MESO-4 (Fig 2C).

### High surface expression of MHC-I on MPM cell lines

**Fig 3:**
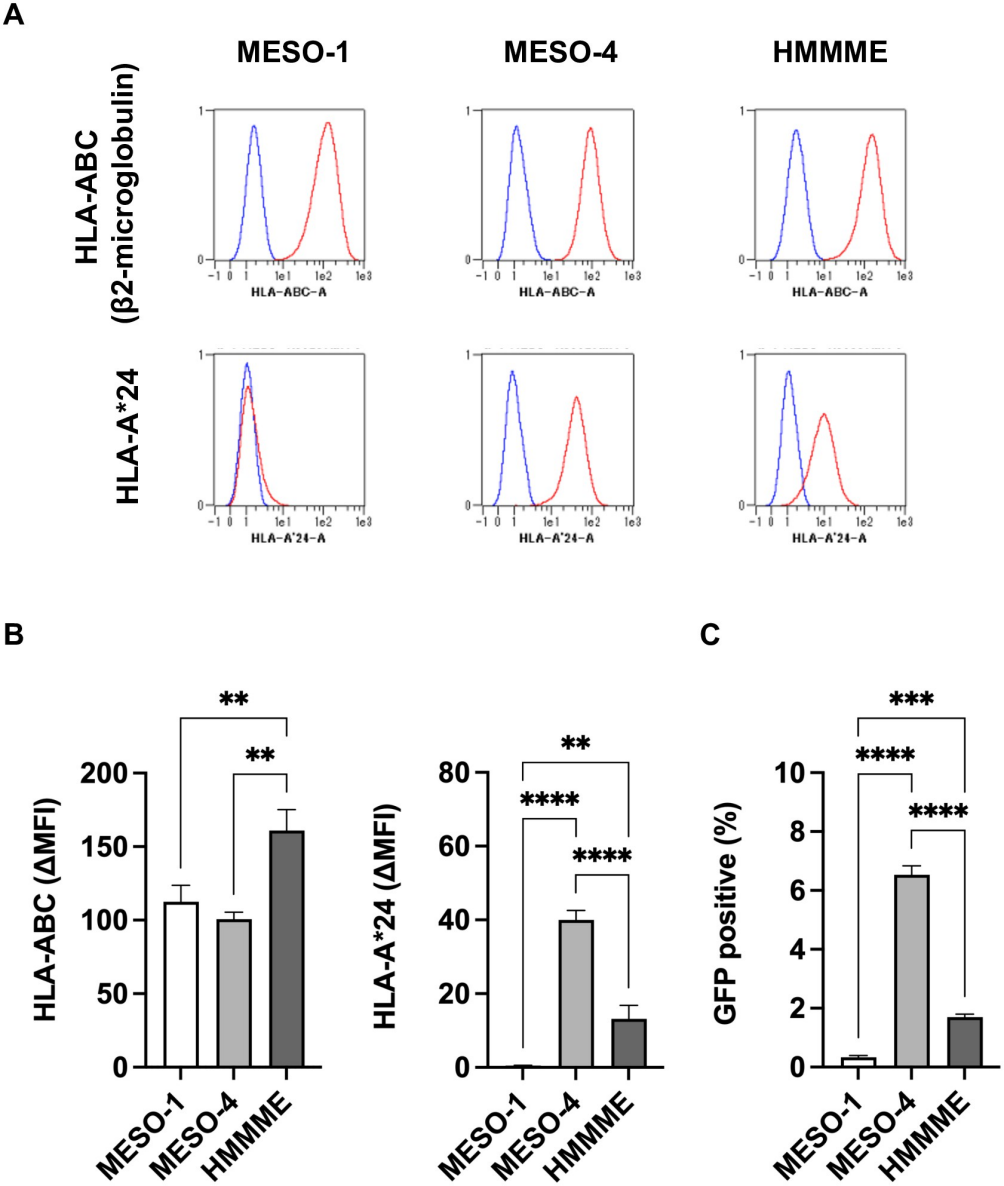
HLA-ABC and HLA-A*24 expression and the cellular immunogenicity of mesothelioma cells. (**A**) Representative flow cytometry histograms of mesothelioma cells stained for the entire HLA-ABC (β2-microglobulin) and HLA-A*24 molecules. Positive cells (red) overlap with the isotype control (blue). (**B**) The bar graph represents the expression of total HLA-ABC and HLA-A*24 on the cell surface. ΔMFI (mean fluorescence intensity (MFI) test-MFI isotype control) is shown. (**C**) The immunogenicity of mesothelioma cells was assessed using an *in vitro* immunogenicity assay. Data are represented as the mean ± SD of three independent experiments and Tukey’s multiple comparisons test was used. **, p<0.01; ***, p<0.001; ****, p<0.0001.

We examined the surface expression of MHC-I on MPM cell lines by flow cytometry. Total MHC-I was assessed by anti-β2-microglobulin presentation by all MPM cell lines (Fig 3A and 3B). Using an anti-HLA-A*24 specific antibody, we found HLA-A*24 expression in MESO-4 and HMMME, but not MESO-1, consistent with the HLA genotype. HLA-A*24 expression was significantly lower in HMMME than MESO-4 (p<0.0001) (Fig 3B). In general, cancer cells have extremely low MHC-I expression [33], but MPM cell lines had high levels of MHC-I, making them suitable target cells for cancer cell immunogenicity assessment.

### WT1-specific cellular immunogenicity of MPM cells is detected by reporter T cells

We investigated whether WT1-specific cellular immunogenicity could be determined using reporter T cells in MPM cell lines. WT1-specific cellular immunogenicity was observed in MESO-4 and HMMME, expressing both HLA-A*24 and WT1, but not MESO-1, which does not express HLA-A*24 (Fig 3C). The strength of WT1-specific immunogenicity was consistent with HLA-A*24 and WT1 antigen expression (Figs 2C and 3B). Different reactivities of reporter cells paralleled the expression levels of WT1 and/or MHC-I molecules. Therefore, we used MESO-4 to search for factors that enhanced cancer cell immunogenicity.

### Immunoproteasome-selective inhibitor ONX-0914 enhances WT1-specific cellular immunogenicity

**Fig 4:**
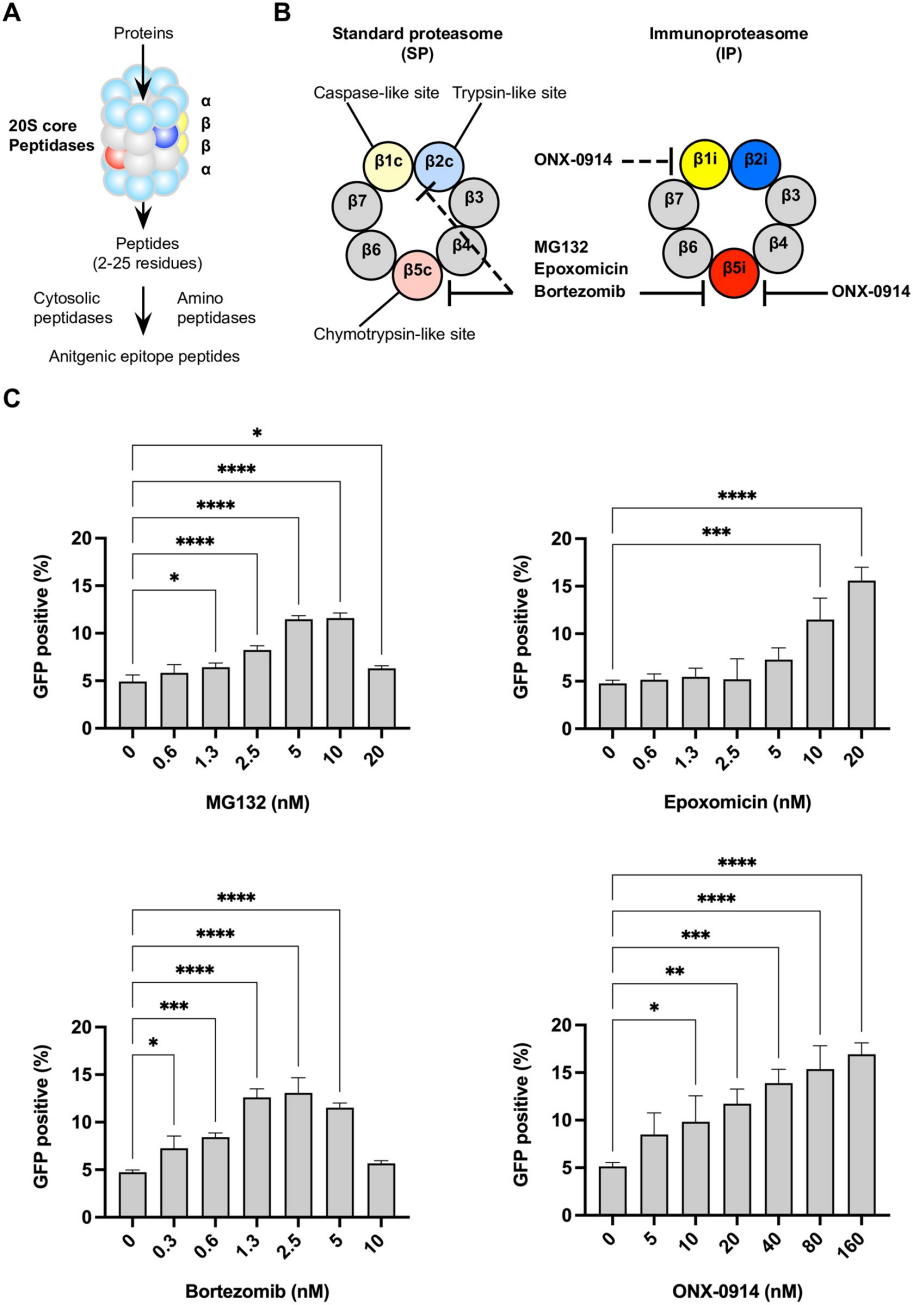
The cellular immunogenicity of mesothelioma is augmented by the immunoproteasome-selective inhibitor ONX-0914. (**A**) Structure of the 20S proteasome core and β-ring. The 20S proteasome core particle comprises four rings of seven subunits: two outer rings of α-subunits and two inner rings of β-subunits. The poly-ubiquitylated proteins are translocated into the 20S core where proteolysis occurs to produce short peptides. Aminopeptidases trim the short peptides to generate antigenic peptides of optimal length for binding to MHC-I molecules. (**B**) Three different β-subunits form a β-ring with different specificity and catalytic properties. Standard proteasome (β1c, β2c, and β5c) and immunoproteasome (β1i, β2i, and β5i) cleave substrates differently, producing different peptides (immunopeptidome). Proteasome inhibitors (MG132, epoxomicin, and bortezomib) mainly inhibit β5c and β5i, but also β1c and β2c. ONX-0914, an immunoproteasome selective inhibitor, mainly inhibits β5i but also β1i. (**C**) MESO-4 was treated with the indicated proteasome inhibitors for 3 hours, then co-cultured with reporter T cells for 20 hours, followed by a cellular immunogenicity assay. Data are represented as the mean ± SD of three independent experiments and Dunnett’s multiple comparisons test was used. *, p<0.05; **, p<0.01; ***, p<0.001; ****, p<0.0001. Representative data results from two independent experiments are shown.

Because proteasomes are critical for the generation of MHC-I epitopes, we investigated the effect of proteasome inhibitors on mesothelial cell immunogenicity. The proteolytic core of the SP has three pairs of active sites, β1c, β2c, and β5c (Fig 4A and 4B). In the IP, these subunits are replaced by subunits with different catalytic activities, termed β1i, β2i, and β5i. Different proteasome subtypes can generate different epitope repertoires based on their enzymatic properties [34–36]. Therefore, we evaluated the cytotoxicity and immunogenicity of four proteasome inhibitors with different specificities (Fig 4B). MG132 is a peptide aldehyde, proteasome, and calpain (cysteine protease) inhibitor [37]. MG132 binds to the chymotrypsin-like site (β5c) and β5i. After 48 hours of treatment, MESO-4 viability was significantly decreased at 100 nM of MG132 (S2 Fig). MG132 at 1.3 nM had enhanced immunogenicity (Fig 4C). However, immunogenicity decreased at 20 nM, probably due to MG132 cytotoxicity. Epoxomicin, a natural epoxyketone that reacts with a chymotrypsin-like site (β5c), is a more selective proteasome inhibitor than MG132 because it does not inhibit cysteine or serine proteases. Epoxomicin had increased immunogenicity at 10 and 20 nM. However, epoxomicin was cytotoxic at 20 nM. Bortezomib (Velcade), a peptide boronate, is a more potent synthetic inhibitor of the proteasome than peptide aldehydes such as MG132 and is used to treat multiple myeloma and mantle cell lymphoma. Bortezomib at 0.3 nM significantly increased MESO-4 immunogenicity, whereas 10 nM bortezomib was toxic and decreased immunogenicity (Fig 4C and S2 Fig). These proteasome inhibitors inhibit β5c and β5i but were reported to co-inhibit other sites (β1c, β2c) at high concentrations [35]. Because these less specific proteasome inhibitors were toxic to MESO-4, we investigated the effect of a more subunit selective proteasome inhibitor (ONX-0914) on immunogenicity. ONX-0914 selectively interacts with the β5i and β2i subunits of the IP (Fig 4B) [36]. Compared with bortezomib, only the highest concentration of ONX-0914 (200 nM) induced cell death after 48 hours of treatment (S2 Fig). Thus, proteasome inhibitors with multiple subunit specificity were more toxic than inhibitors with higher subunit selectivity. ONX-0914 enhanced MESO-4 immunogenicity at 10 nM (Fig 4C). Between 10–160 nM, ONX-0914 enhanced immunogenicity dose-dependently. The proteasome inhibitors used in this experiment demonstrated increased immunogenicity. All four drugs tested inhibit IP subunit β5i functions [35, 36]. ONX-0914, which does not inhibit SP, significantly increased immunogenicity. Thus, enhanced WT1-specific immunogenicity of MESO-4 was associated with suppressed IP function.

Additionally, experiments were performed to demonstrate that ONX-0914 has the unique capacity to enhance immunogenicity against the WT1_235_ epitope. An artificial protein antigen, designated WT1-242, was created by incorporating WT1_235_ epitopes and α-helix structures to stabilize the protein structure. This antigen was then transfected into 293-A*24 cells, which expressed HLA-A*24 (S3A and S3B Fig). Expression of the transcription factor WT1 full-length cDNA may affect the phenotype of 293-A*24 cells, but the use of this WT1-242 artificial antigen will eliminate the possibility of this phenotypic change. The treatment of WT1-242-expressing 293-A*24 cells with ONX-0914 resulted in enhanced immunogenicity for reporter T cells (S3C Fig). These results suggest that ONX-0914 can enhance the immunogenicity of the WT1_235_ epitope specifically.

Furthermore, an increase in the immunogenicity of the WT1_235_ epitope was observed in 293-A*24 cells, in which the expression of the mature β5i molecule was reduced (S4 Fig). These results indicate that ONX-0914 enhances the immunogenicity of the WT1_235_ epitope by inhibiting the function of β5i.

### Immunoproteasome-selective inhibitor ONX-0914 does not affect WT1 and MHC-I expression

To determine whether ONX-0914 confers enhanced immunogenicity to MESO-4 by increasing the expression of WT1 antigen, we examined the expression level of WT1 in MESO-4 by western blotting (S5A and S5B Fig). ONX-0914 had no effect on the expression of WT1 protein.

We also examined the effect of ONX-0914 on the expression of MHC-I molecules. The expressions of HLA-A*24 and HLA-ABC molecules were not affected by ONX-0914 (S5 Fig). These results suggest that the enhancement of WT1-specific immunogenicity by ONX-0914 treatment is not related to increased WT1 antigen expression and MHC-I expression.

### ONX-0914 does not affect the expression of immunoproteasome subunits β5i and β1i

ONX-0914 primarily binds to β5i but has also been reported to bind to β1i [35]. Therefore, we examined the effect of ONX-0914 on β1i and β5i expressions (S5A and S5B Fig). Treatment of MESO-4 with 20 nM ONX-0914 for 3 hours altered β5i expression, but not β1i expression. This result suggests that ONX-0914 selectively binds to β5i. IFNγ is a known inducer of IP expression. Therefore, we investigated whether treatment with 10 ng/ml IFNγ for 3 hours would affect the expressions of β1i and β5i, but no change was observed in MESO-4. β1i and β5i mRNA expression levels were examined by semiquantitative PCR (S5C and S5D Fig). The expressions of β1i and β5i mRNA were increased by IFNγ treatment, but ONX-0914 had no effect on their expression (S5C and S5D Fig). Treatment of MESO-4 with 20 nM ONX-0914 for 3 hours enhanced WT1 immunogenicity (Fig 4C), but such low concentrations of ONX-0914 did not induce β1i or β5i mRNA expressions. Taken together, these results suggest that the enhancement of WT1-specific immunogenicity by ONX-0914 in MESO-4 cannot be explained by an increase or decrease in β1i and β5i expressions.

### ONX-0914 binds selectively to the β5i catalytic subunits of the IP

**Fig 5:**
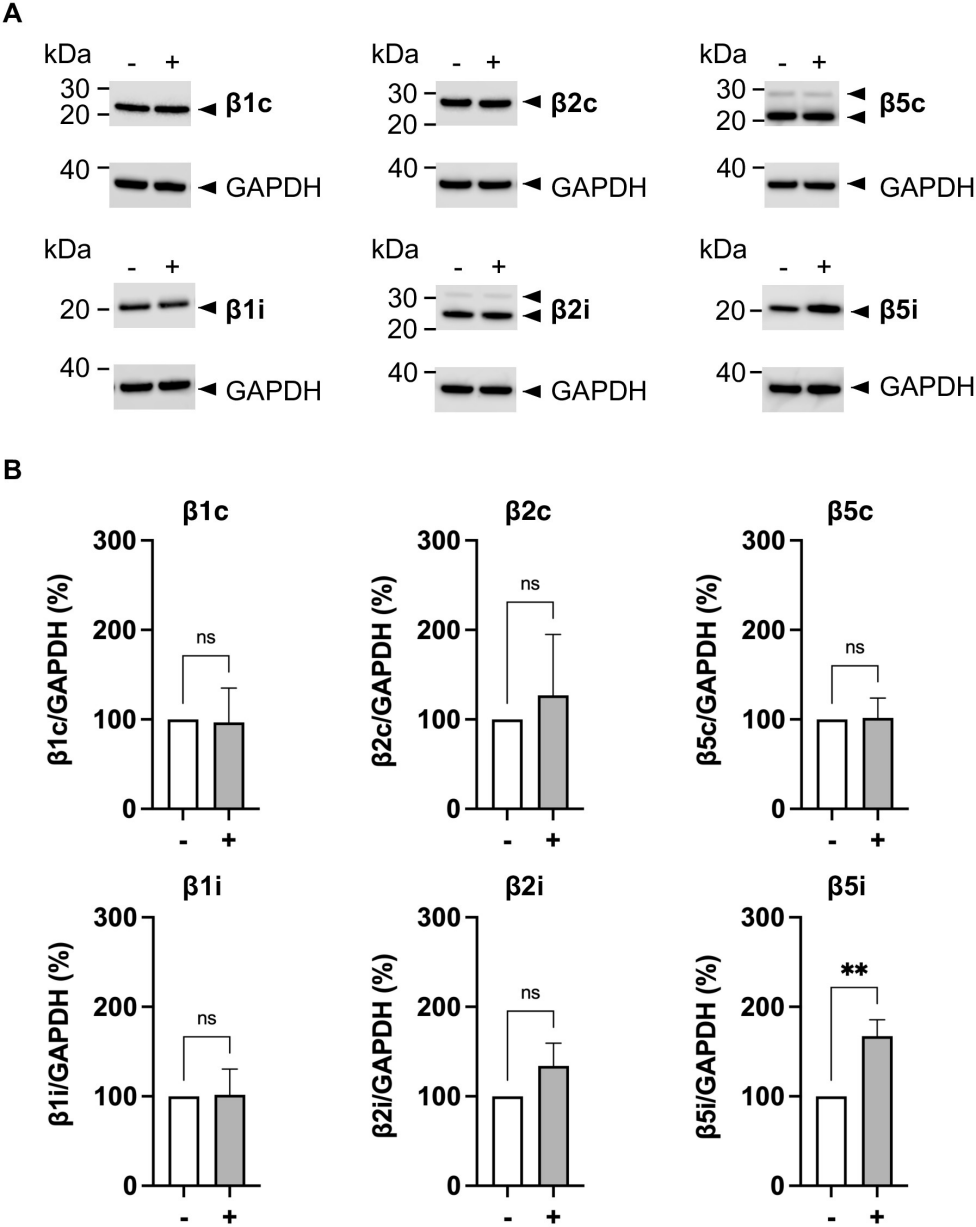
Expression of 20S proteasome β-subunits treated with ONX-0914, a selective inhibitor of the immunoproteasome. (**A**) Representative western blots of protein extracts from MESO-4 treated with 20 nM ONX-0914 for 3 hours (indicated by +). Protein expressions of β1c, β2c, β5c, β1i, β2i, and β5i were analyzed by western blotting using the indicated antibodies. (**B**) Quantification of 20S proteasomal β-subunit expression was analyzed by western blotting and values were normalized to control (GAPDH). Data are represented as the mean ± SD of three independent experiments and an unpaired t-test was used. ns, not significant; **, p<0.01.

The proteolytic core of the SP in all eukaryotic cells has three pairs of active sites, β1c, β2c, and β5c (Fig 4B). IP is expressed in hematopoietic stem cells as well as cancer cells. When cancer cells are exposed to IFNγ released by T cells, the SP subunits (β1c, β2c, β5c) are replaced by IP subunits (β1i, β2i, β5i). Therefore, we investigated the expression of SP and IP catalytic β-subunits in MESO-4. In addition to SP, all the catalytic β-subunits (β1i, β2i, and β5i) of IP were expressed endogenously in MESO-4 (Fig 5A and 5B). ONX-0914 increased the intensity of the β5i subunit band, and no change was observed for the other subunits. These results suggest that ONX-0914 treatment selectively inhibits the function of the β5i subunit of the immunoproteasome in MESO-4.

### ONX-0914 improves the immunogenicity of HLA-A*24:02 transduced MESO-1 and HMMME

We investigated whether ONX-0914 improved cellular immunogenicity in mesotheliomas other than MESO-4. Reporter cells did not react with MESO-1 lacking HLA-A*24:02, but ONX-0914 improved immunogenicity in MESO-1 transfected with the HLA-A*24:02:01 gene and in HMMME (S7A and S8 Figs). Western blotting confirmed that HLA-A*24:02:01 transduced MESO-1 and HMMME also expressed β5i (S7B Fig). In addition, treatment with ONX-0914 resulted in a band shift of β5i, suggesting that ONX-0914 binds to β5i (S7B Fig). These results suggest that ONX-0914 enhances the WT1_235_-specific immunogenicity of mesothelioma cells expressing both WT1 and IP.

### ONX-0914 pre-treated MESO-4 are susceptible to WT1-reactive cytotoxic T-cell killing

**Fig 6:**
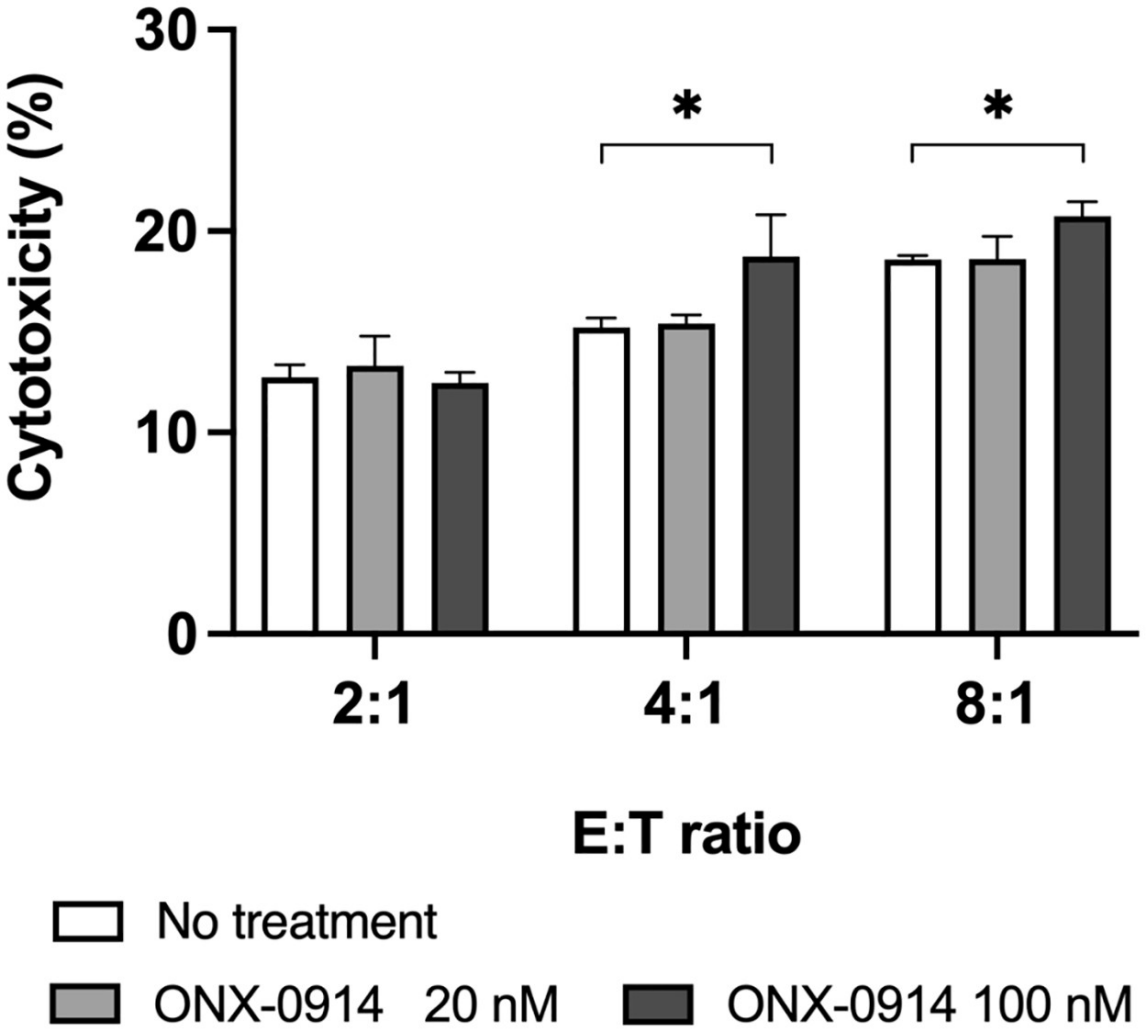
MESO-4 pre-treated with ONX-0914 showed an increase in sensitivity to cytotoxic T cells. ONX-0914-treated MESO-4 (1x10^5^) and CD8-positive T cells, including WT1-specific cytotoxic T cells, were co-cultured at the ratios shown in the graph. Cytotoxicity was assessed by LDH assay. Data are represented as the mean ± SD of three independent experiments and Dunnett’s multiple comparisons test was used. *, p<0.05. Representative data results from two independent experiments are shown.

To determine whether ONX-0914 sensitized MESO-4 to WT1-reactive cytotoxic T cells (WT1-CTLs), an LDH cytotoxicity assay was performed. CD8-positive cells containing 6% WT1-specific CTL induced in previous clinical trials were used as WT1-CTLs [28, 29]. MESO-4 pre-treated with ONX-0914 at 100 nM were susceptible to killing by WT1-CTLs (Fig 6). These results suggest that ONX-0914 increases the amount of WT1 epitope presented to MHC-I molecules on MESO-4.

### ONX-0914 prevents internal destructive cleavage of the WT1_235_ epitope by IP

**Fig 7:**
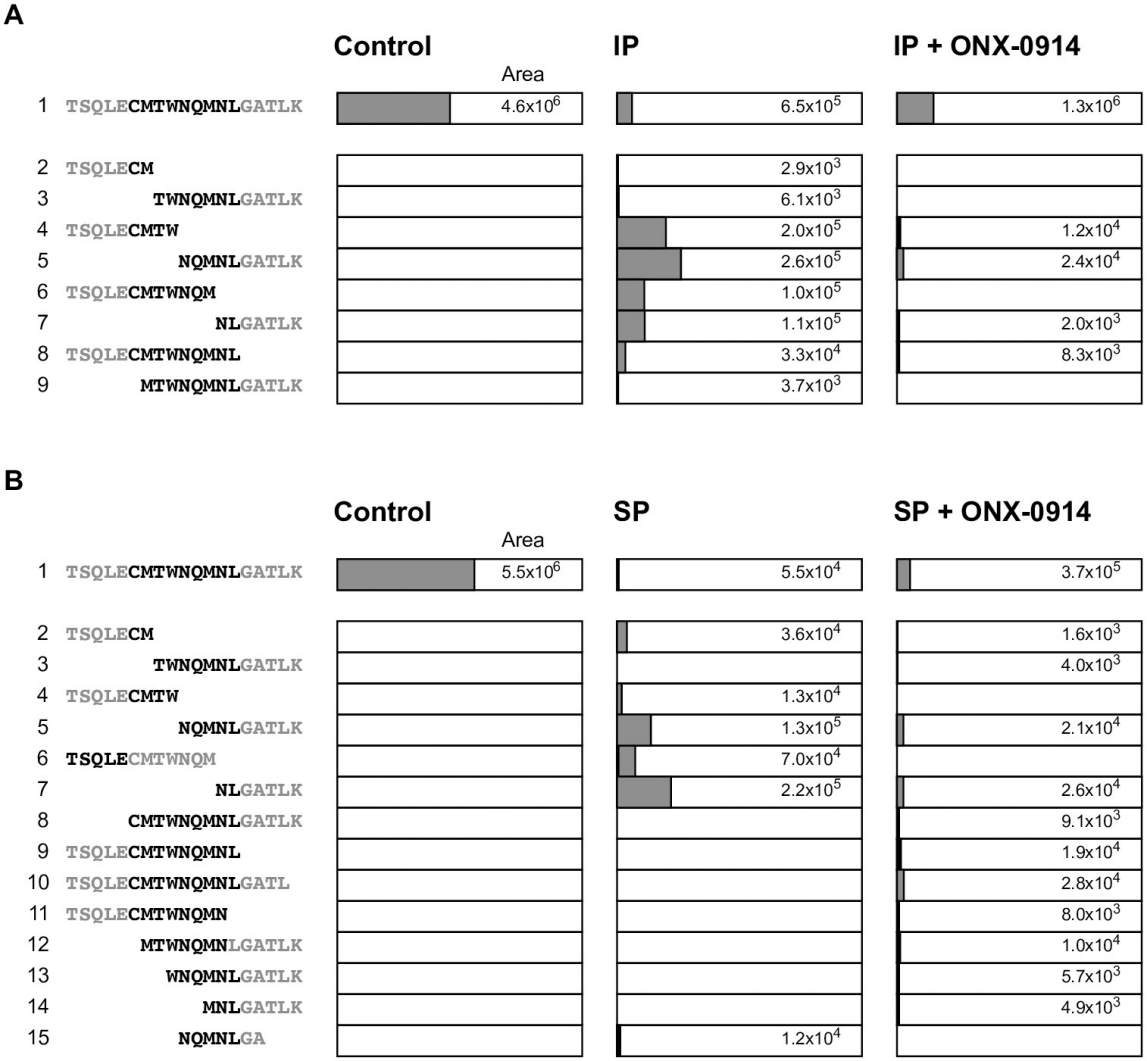
IP causes internal destructive cleavage of the WT1_235_ epitope. Precursor peptides containing the WT1_235_ epitope were digested with purified immunoproteasome (IP) (**A**) and standard proteasome (SP) (**B**) with and without ONX-0914 for 3 hours. Proteolysis products were evaluated by tandem mass spectrometry to measure production efficiency. Each fragmented peptide peak area was determined using extracted peak chromatograms. WT1_235_ epitope fragments are highlighted by black text. Analysis is representative of two independent experiments.

As described above, the enhancement of WT1_235_-specific immunogenicity by ONX-0914 in mesothelial cells cannot be explained by quantitative changes in MHC-I, WT1, or IP expression levels. Therefore, we investigated the possibility that internal cleavage of the WT1 antigen epitope occurs in MESO-4. Previously, Chapiro and colleagues reported that the melanoma antigen peptide tyrosinase_369_ (YMDGTMSQV) was digested to a greater degree by IP than by SP, which cleaves the peptide after an internal hydrophobic residue and is less immunogenic to tyrosinase-specific CTLs [22]. We found that the WT1_235_ sequence had two hydrophobic methionine residues similar to the tyrosinase_369_ sequence. Therefore, we performed *in vitro* proteasome digestion assays and mass spectrometry analysis using a precursor peptide (TSQLE**CMTWNQMNL**GATLK) containing the WT1_235_ epitope to investigate the mechanism of immunogenicity enhancement of ONX-0914. The precursor peptide was cleaved by IP mainly after tryptophan (W) and methionine (M), which are hydrophobic amino acids within the WT1_235_ epitope (Fig 7A). In addition, this internal destruction of the WT1_235_ precursor peptide by the IP was inhibited by ONX-0914.

Interestingly, ONX-0914 also suppressed the internal cleavage of WT1_235_ in SP (Fig 7B). In MESO-4 with persistent IP expression, internal destructive cleavage of the WT1_235_ epitope may occur. As a result, the amount of WT1_235_ epitope presented to the MHC-I molecule may be reduced. These results suggest that ONX-0914 suppresses IP-induced internal cleavage of the WT1_235_ epitope and enhances WT1-specific immunogenicity of MESO-4.

## Discussion

To date, cancer immunotherapy has focused primarily on the ability to induce cancer antigen-specific CTLs. However, the dramatic anti-tumor effects of immune checkpoint inhibitors have shown that in cancer patients, CTLs that attack cancer cells are already induced *in vivo*, and immune checkpoints induce CTL exhaustion, resulting in immune escape by cancer cells [38, 39]. This finding highlights the limitations of development strategies such as conventional vaccines for cancer immunotherapy, which are primarily focused on the induction of CTLs. Because it is already possible to reactivate exhausted CTLs with immune checkpoint inhibitors, this study focused on increasing the sensitivity of cancer cells to CTLs. Therefore, we sought to identify compounds that would increase cancer cell immunogenicity.

MHC-I and antigen expression are key determinants of cell immunogenicity. Hematopoietic cells such as dendritic cells and lymphocytes always express high levels of MHC-I, but epithelial cells in normal tissues do not express MHC-I to the same extent as hematopoietic cells [40]. It was reported that MHC-I antigen presentation is downregulated in cancer cells of epithelial origin by abnormalities in the antigen processing machinery [41], which include MHC-I heavy chains, β2M, immunoproteasome subunits, TAP, Tapasin, and ERAP1. Cancer cells also showed a reduced expression of dominant antigen and neoantigen [41]. Therefore, to identify factors that influenced the immunogenicity of cancer cells, we decided to use mesothelioma cells as target cancer cells, in which MHC-I and the target antigen WT1 are highly expressed.

In cells of mesodermal origin, such as blood cells and mesothelium, WT1 is expressed at high levels [4–6]. Indeed, all mesothelioma cell lines of mesothelial origin examined in this study expressed high levels of WT1 (Fig 2). When antigen presentation of the HLA-A*24 restricted WT1_235_ epitope was measured, reporter T-cell activation was stronger in MESO-4 with higher HLA-A*24 expression and higher WT1 antigen expression than in HMMME. These results confirmed that MHC-I and antigen expression are important determinants of cellular immunogenicity.

As a factor affecting cellular immunogenicity, we focused on the proteasome, which regulates the generation of immunogenic epitope peptides from protein antigens. All four proteasome inhibitors used promoted increased immunogenicity. However, MG132, epoxomicin, and bortezomib exhibited cytotoxicity at low concentrations (S2 Fig). Inhibitors with low proteasome subunit specificity were reported to be highly cytotoxic to cells with SP and IP expression [42]. Indeed, the IP-selective inhibitor ONX-0914, which binds selectively to β5i, was less toxic than other proteasome inhibitors (S2 Fig). In addition, no significant adverse effects have been reported in animal studies following the administration of ONX-0914 [42–44]. ONX-0914 improved the symptoms of autoimmune diseases and ulcerative colitis and has immunomodulatory properties [43, 44]. Koerner and colleagues reported that ONX-0914 suppressed tumor development in chemically-induced azoxymethane/dextran sodium sulfate and transgenic (Apc^min/+^) mouse models of colon carcinogenesis [45]. Those studies proposed that ONX-0914 had anti-inflammatory effects mediated by suppressing the production of IL-6 and IL-17 cytokines. Large numbers of dendritic cells expressing high levels of IP present in the colon constantly monitor bacterial invasion. Thus, ONX-0914 might act on dendritic cells to modulate antigen presentation, thereby suppressing inflammation in the colon and potentially inhibiting carcinogenesis. Alternatively, it may have a direct effect on colon tumors to suppress carcinogenesis by modifying the immunogenicity of tumor cells. Future animal studies are needed to determine whether ONX-0914 also has anti-tumor activity in mesothelioma, which is caused by long-term inflammation related to asbestos exposure.

Our study suggests that the enhancement of WT1-specific immunogenicity of mesothelioma cells by ONX-0914 was due to the inhibition of the internal disruptive cleavage of the WT1_235_ epitope peptide. As a result, the amount of WT1_235_ epitope presented to MHC-I was increased, which may have enhanced the WT1-specific immunogenicity of MESO-4.

Chapiro and colleagues previously reported the destructive cleavage of antigenic peptides by the IP or SP resulted in different antigen presentations [22]. They found that the tyrosinase_369_ sequence (YMDGTMSQV), a melanoma antigen, was cleaved internally by IP behind an internal hydrophobic methionine residue. We observed that WT1_235_ (CMTWNQMNL) had two internal methionine residues, similar to tyrosinase_369_. An *in vitro* digestion assay showed that the internal cleavage of the peptide by IP actually occurred behind the methionine and tryptophan residues, which are hydrophobic amino acids in WT1_235_ (Fig 7A). In addition, a functional T cell reporter assay showed that ONX-0914 enhanced the immunogenicity of WT1_235_ dose-dependently (Fig 4C). These results suggest that internal cleavage of the WT_235_ epitope by IP occurred in MESO-4 that continuously expressed both IP and WT1 antigen. The IP subunit β5i was expressed in all three types of mesothelioma cells tested in this study, and the binding of ONX-0914 to β5i was also confirmed (S2 and S7B Figs). Although the role of IP in mesothelioma is still unknown, the combination of T-cell transfer therapy targeting WT1_235_ with an IP subunit inhibitor such as ONX-0914 may improve therapeutic efficacy in mesothelioma.

In the WT1_235_ epitope, we found that the SP was also cleaved after the hydrophobic amino acids methionine and tryptophan (Fig 7B). This cleavage was inhibited by ONX-0914. It is unclear whether this was related to the purity of SP used in this experiment or the specificity of ONX-0914. In addition, it is conceivable that the immunoproteasomes other than β5i, standard proteasomes, and aminopeptidases may also influence the immunogenicity-enhancing effects of ONX-0914.

Guillaume and colleagues reported the existence of an intermediate proteasome (IMP) between the SP and IP containing β5i and that the internal destructive cleavage of tyrosinase_369_ also occurred in this hybrid form [16]. Future studies should determine whether IMP between SP and IP are also functional in MESO-4.

Moreover, ONX-0914 may not only selectively inhibit β5i, but also cause abnormal proteolysis and the generation of new immunogenic peptides. It is also essential to investigate the impact of ONX-0914 on cellular immunogenicity other than the internal destructive cleavage of the WT1 epitope.

Jaigirdar and colleagues investigated the immunogenicity of cells using TCR-transfected T cells that recognized the WT1_126_ (RMFPNAPYL) epitope, which binds to HLA-A*02:01 [20]. They reported that when WT1 was expressed in 293 cells transfected with SP or IP, the 293 cells with IP had a higher level of immunogenicity. Lahman and colleagues showed that the β1i subunit of the IP was required for antigen presentation of the WT1_126_ epitope [21]. WT1_126_ also contains a hydrophobic methionine residue at position 2, but may not have been internally degraded by IP. They also identified a novel HLA-A*02-binding WT1_37_ epitope (VLDFAPPGA) that was immunogenic in an IP-independent manner. The amino acid sequence of the protein determines which epitopes are produced from the antigen by the proteasome. When selecting targets for TCR-delivered T-cell therapy, the amino acid sequence of the antigen and the proteasome composition within the cell, which affects immunogenicity, should be considered.

MESO-4 expressing multiple SPs and IPs are suitable for the assessment of cell immunogenicity. TCRs that recognize a variety of WT1 epitopes that are antigenically presented on HLA-A*24 and HLA-A*02 have been identified [13, 14, 21, 25]. HMMME express HLA-A*02. The specificity of the TCR could be more accurately assessed if the reactivity of the WT1 epitope-specific TCR was assessed using mesothelioma cells expressing MHC-I, WT1 antigen, and IP, such as MESO-4 and HMMME.

The development of cancer immunotherapies targeting neoantigens is being pursued vigorously. In this strategy, mRNA and genomic information are first used to identify neoantigens that are primarily responsible for amino acid missense mutations. Candidate neoantigen epitopes that bind to MHC-I molecules are then identified by algorithmic analysis and the binding of the peptides to MHC-I molecules is functionally verified using an *in vitro* T2 cell assay [46]. The identification of neoantigens in melanoma was described in detail by Carreno and colleagues. They report that among the neoantigens identified by rigorous analysis, there were a small number of epitopes that were not immunogenic when forced expression was finally achieved in melanoma cells [46]. As shown in this study, the processing of antigens by the proteasome may be responsible for the reduced immunogenicity of neoantigens in cancer cells. For the future development of immunotherapies, in addition to confirming the expression of neoantigens in target cells, it will be necessary to assess how neoantigens are processed by the proteasome of target cells.

Immune checkpoint inhibitors are already in use for mesothelioma treatment [2]. However, if the immunopeptidome presented by MHC-I can be altered by the use of selective immunoproteasome inhibitors such as ONX-0914, the type and amount of neoantigen-derived epitopes other than WT1 may be altered and the immunogenicity of mesothelioma cells may be enhanced. The combination of immune checkpoint inhibitors and selective immunoproteasome inhibitors is expected to have a potent antitumor effect, resulting in increased cytotoxic T cells that target neoantigens in mesothelioma cells.

In this study, we showed that selective inhibitors of the immunoproteasome had an immunomodulatory effect on mesothelioma cells and controlled their immunogenicity. In future studies, the anti-tumor effects of selective immunoproteasome inhibitors will be evaluated *in vivo*, alone and in combination with immune checkpoint inhibitors.

## Supporting information

S1 Fig Flow cytometry gating for activated reporter T cells. After reporter T cells (B10-TCR td 2D3 cell line) were co-cultured with target cells, debris and doubles were removed, then 7-AAD^**-**^/CD8^**+**^ cells were gated. Reporter T cells permanently expressing Venus fluorescent protein induce GFP by activation when cells are treated with phorbol 12-myristate 13-acetate (PMA) + ionomycin. The activated reporter T cells were determined based on GFP and Venus double-positive cells.
(TIF)

S2 Fig Cytotoxicity of the immunoproteasome-selective inhibitor ONX-0914 to MESO-4 is lower than MG132, epoxomicin, and bortezomib. Cells were treated with various concentrations of proteasome inhibitors for 48 hours, followed by the measurement of cell viability using Cell Count Reagent SF (Nacalai Tesque, #07553–44). The immunoproteasome-specific inhibitor ONX-0914 does not show cytotoxicity at a concentration of 20 nM. Data are represented as the mean ± SD of three independent experiments and Dunnett’s multiple comparisons test was used. ***, p<0.001; ****, p<0.0001.
(TIF)

S3 Fig The reporter T cells (B10-TCR td 2D3 cell line) are capable of recognizing the immunogenicity of the WT1_235_ epitope in a specific manner. WT1-242, a WT1 artificial antigen consisting of the WT1_235_ epitope and an α-helix structure stabilizing the protein, was expressed in HLA-A*24 expressing 293 cells to investigate the specificity of the reporter T cells. (**A**) Representative western blots were used to probe HA-tag, Myc-tag, WT1, β5c, β5i, and GAPDH in 293 HLA-A*24 cells treated with 100 nM of ONX-0914 for 20 hours (indicated by +). 1: 293-A24 cells, 2: WT1-242-expressing 293-A24 cells, 3: MESO-4 cells. **(B**) The amino acid sequence of WT1-242. (**C**) The activity of reporter T cells was specifically confirmed to be enhanced by WT1-242 expression. Furthermore, the immunogenicity of the cells was enhanced by ONX-0914 treatment. Data are represented as the mean ± SD of three independent experiments and Dunnett’s multiple comparisons test was used. ****, p<0.0001.
(TIF)

S4 Fig A deficiency of β5i enhances the immunogenicity of the WT1_235_ epitope. 293-A*24 cells deficient in β5i (β5i-KO) were generated in accordance with the manufacturer’s instructions (Invitrogen, #A35534, PSMB8_C1). (**A**) Representative western blots were used to probe β5i and GAPDH in 293-A*24 and β5i-KO cells. **(B**) A reduction in the level of mature β5i expression in 293-A*24 cells has been observed to enhance the activity of reporter T cells. Data are represented as the mean ± SD of three independent experiments and Unpaired t test was used. **, p<0.01.
(TIF)

S5 Fig The immunoproteasome-selective inhibitor ONX-0914 binds to the β5i subunit. (**A**) Representative western blots were used to probe WT1, β1i, β5i, and GAPDH in MESO-4 treated with ONX-0914 (20 nM) and IFNγ (10 ng/ml) for 3 hours. (**B**) Densitometry values of expression relative to GAPDH are indicated. Increased expression of β5i protein by treatment with ONX-0914 indicates that ONX-0914 is mainly bound to the β5i protein. (**C**) A representative gel shows a typical RT-PCR analysis of β1i (322 bp) and β5i (172 bp) mRNAs with GAPDH (540 bp) as the internal control in MESO-4 treated with ONX-0914 (20 nM) and IFNγ (10 ng/ml) for 3 hours. RT-PCR products were analyzed on a 2.0% MetaPhor^™^ agarose gel. (**D**) The bar graph represents the results of the quantitative analysis of β1i and β5i mRNA levels normalized against GAPDH. MESO-4 treated with IFNγ had significantly upregulated β1i and β5i mRNAs but did not change with ONX-0914 treatment. Data are represented as the mean ± SD of three independent experiments and Tukey’s multiple comparisons test was used. ns, not significant; *, p<0.05; **, p<0.01.
(TIF)

S6 Fig The expression of MHC-I molecules is not affected by ONX-0914. Flow cytometric analysis results showing MESO-4 cell surface expression of total MHC-I (β2-microglobulin) and HLA-A*24. ΔMFI (mean fluorescence intensity (MFI) test-MFI isotype control) is shown. Data are represented as the mean ± SD of three independent experiments and Tukey’s multiple comparisons test was used. ns, not significant.
(TIF)

S7 Fig ONX-0914 enhances the WT1_235_ epitope peptide-specific immunogenicity of three different mesothelioma cells. (**A**) MESO-1 expressing HLA*A24 are immunogenic to reporter T cells, and ONX-0914 treatment (indicated by +) further enhances their immunogenicity. Data are represented as the mean ± SD of three independent experiments and an unpaired t-test was used. (**B**) β5i immunoproteasome subunit expression was analyzed by western blotting. Mesothelioma cells were treated with 100 nM ONX-0914 for 3 hours (indicated by +), and the protein expression of β5i was analyzed by western blotting. Treatment with ONX-0914 resulted in a band shift of β5i, suggesting that ONX-0914 binds to β5i.
(TIF)

S8 Fig Establishment of ACC-MESO-1 expressing HLA-A*24:02:01 (MESO-1-A24). Human HLA-A*24:02:01 cDNA was provided by RIKEN BRC through the National BioResource Project of the MEXT, Japan (cat. RDB02871) (1). HLA-A*24:02:01 cDNA was excised using *Nhe*I and *Not*I restriction enzymes and cloned into a PB514B-2 vector (SBI System Bioscience). The cDNA vector and PB210PA-1 super PiggyBac transposase expression vector were introduced into MESO-1 using Nucleofector I (LONZA, Kit V, program T-20). HLA-A*24 expressing MESO-1 (MESO-1-A24) was selected and established in RPMI medium containing 10% FBS supplemented with 0.4 μg/ml of puromycin. The expression of HLA-A*24 was confirmed by flow cytometry using an anti-HLA-A*24 antibody (MBL, clone 22E1, # K0209-5).
Reference
1. Akatsuka Y, Goldberg TA, Kondo E, Martin EG, Obata Y, Morishima Y, *et al*. Efficient cloning and expression of HLA class I cDNA in human B-lymphoblastoid cell lines. Tissue Antigens **2002**;59(6):502–11.
(TIF)

S9 Fig Western blot raw data. (PDF)
