# Supplementary figures and images for: Enhancing the immunogenicity of Wilms tumor 1 epitope in mesothelioma cells with immunoproteasome inhibitors
Source: PLoS One. 2024 Aug 8;19(8):e0308330. doi: 10.1371/journal.pone.0308330 (PMC11309442; doi:10.1371/journal.pone.0308330)

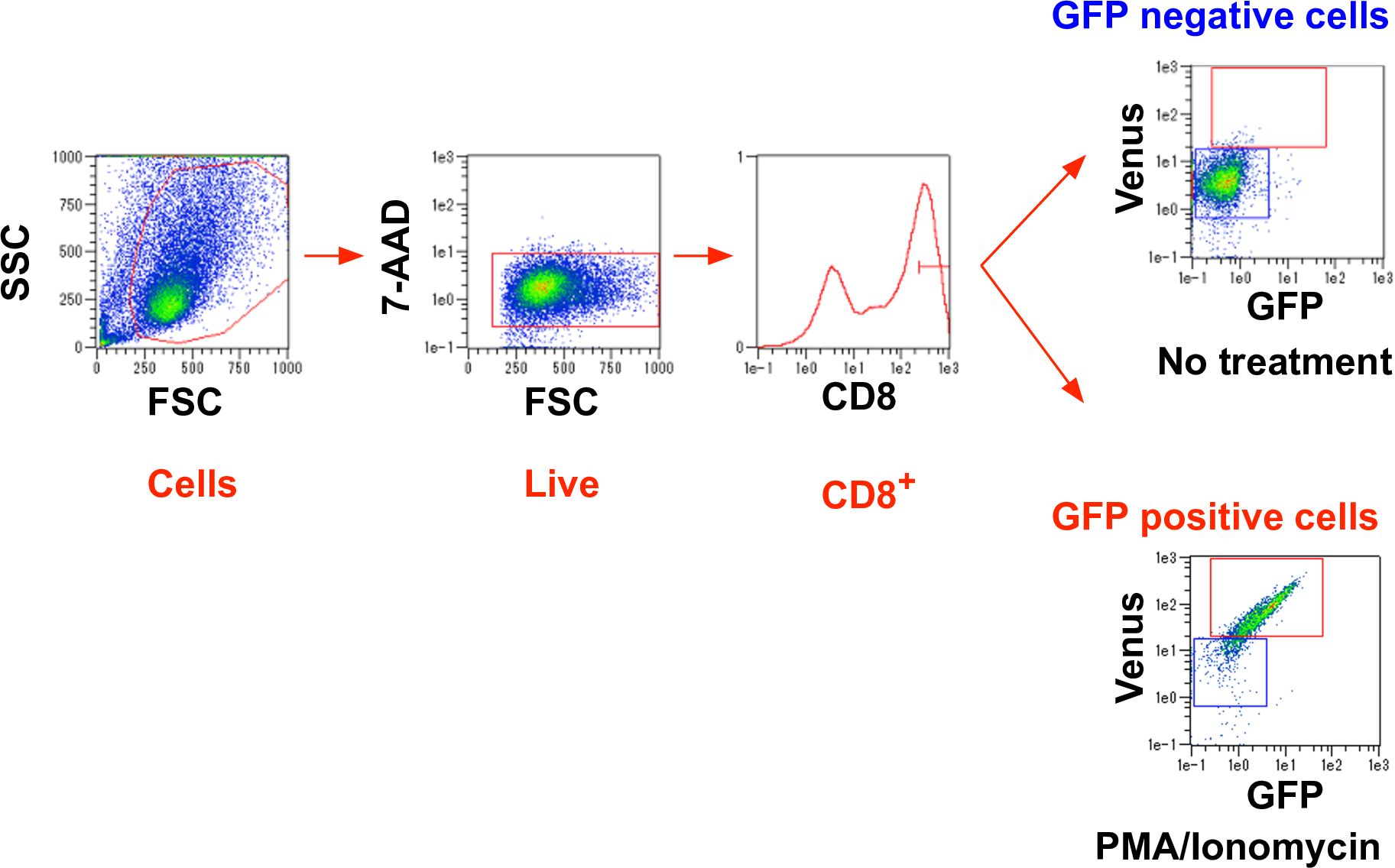

Supplement: S1 Fig — After reporter T cells (B10-TCR td 2D3 cell line) were co-cultured with target cells, debris and doubles were removed, then 7-AAD-/CD8+ cells were gated. Reporter T cells permanently expressing Venus fluorescent protein induce GFP by activation when cells are treated with phorbol 12-myristate 13-acetate (PMA) + ionomycin. The activated reporter T cells were determined based on GFP and Venus double-positive cells. (TIF) [file pone.0308330.s001.tif]

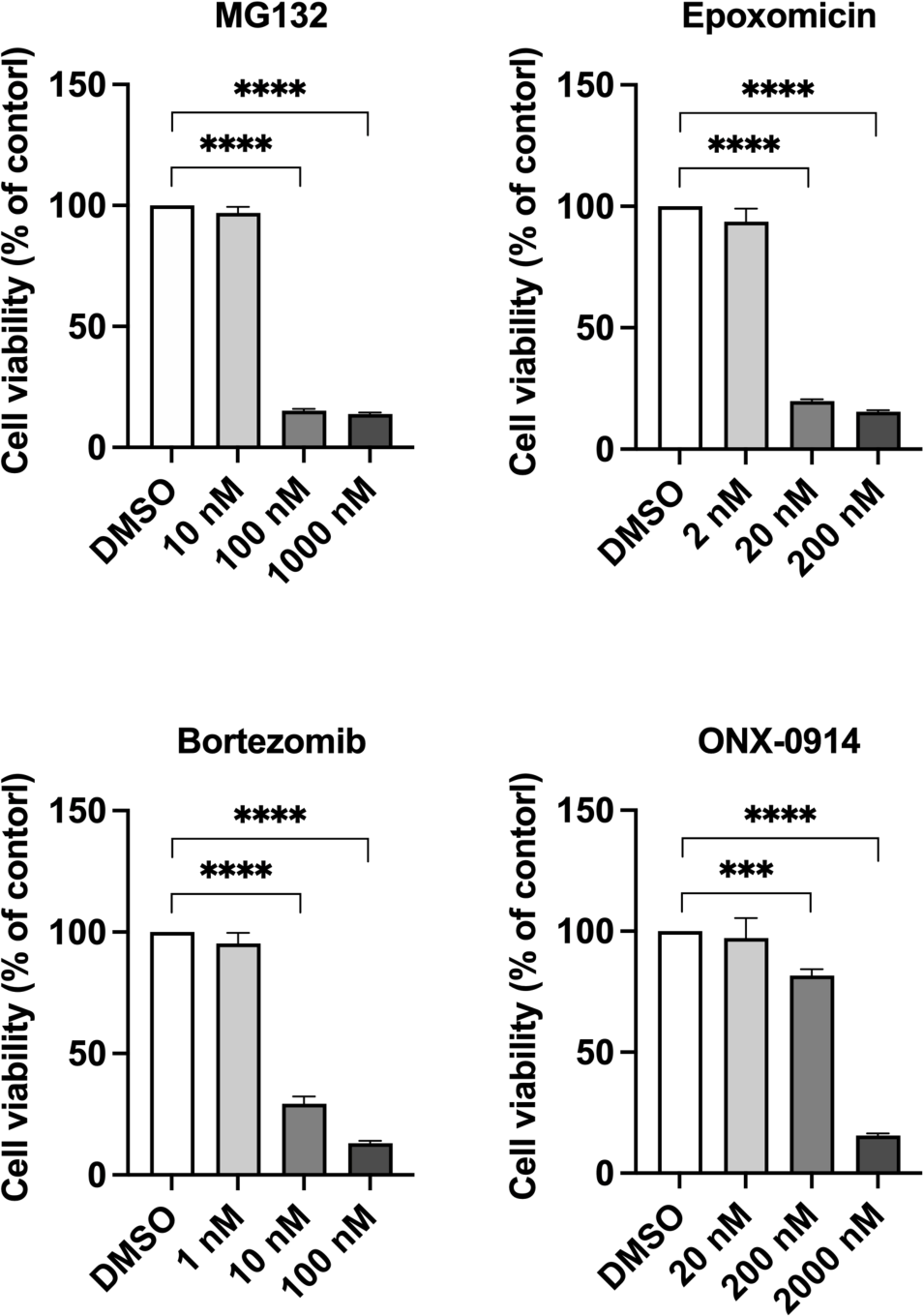

Supplement: S2 Fig — Cells were treated with various concentrations of proteasome inhibitors for 48 hours, followed by the measurement of cell viability using Cell Count Reagent SF (Nacalai Tesque, #07553–44). The immunoproteasome-specific inhibitor ONX-0914 does not show cytotoxicity at a concentration of 20 nM. Data are represented as the mean ± SD of three independent experiments and Dunnett’s multiple comparisons test was used. ***, p<0.001; ****, p<0.0001. (TIF) [file pone.0308330.s002.tif]

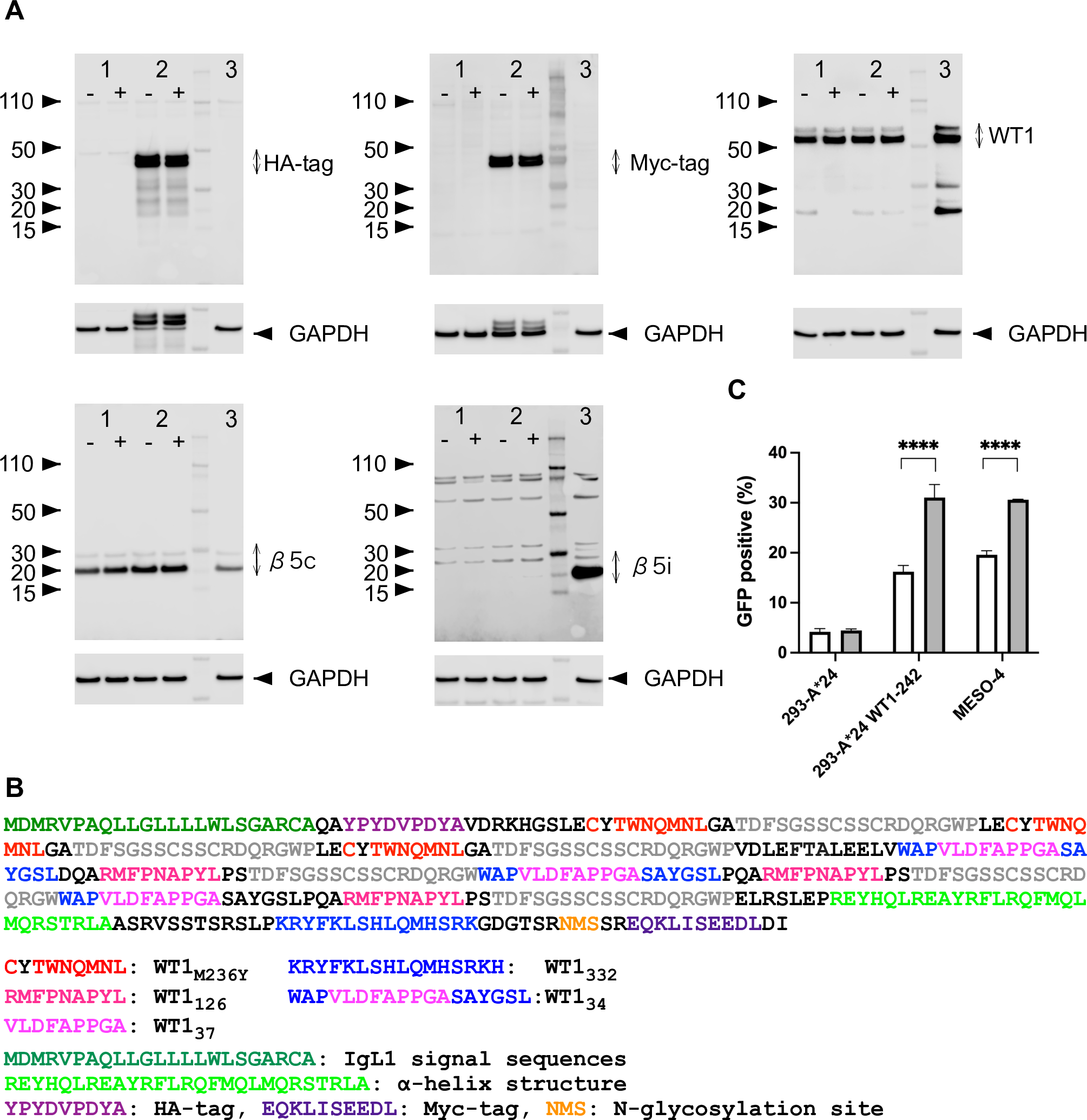

Supplement: S3 Fig — WT1-242, a WT1 artificial antigen consisting of the WT1235 epitope and an α-helix structure stabilizing the protein, was expressed in HLA-A*24 expressing 293 cells to investigate the specificity of the reporter T cells. (A) Representative western blots were used to probe HA-tag, Myc-tag, WT1, β5c, β5i, and GAPDH in 293 HLA-A*24 cells treated with 100 nM of ONX-0914 for 20 hours (indicated by +). 1: 293-A24 cells, 2: WT1-242-expressing 293-A24 cells, 3: MESO-4 cells. (B) The amino acid sequence of WT1-242. (C) The activity of reporter T cells was specifically confirmed to be enhanced by WT1-242 expression. Furthermore, the immunogenicity of the cells was enhanced by ONX-0914 treatment. Data are represented as the mean ± SD of three independent experiments and Dunnett’s multiple comparisons test was used. ****, p<0.0001. (TIF) [file pone.0308330.s003.tif]

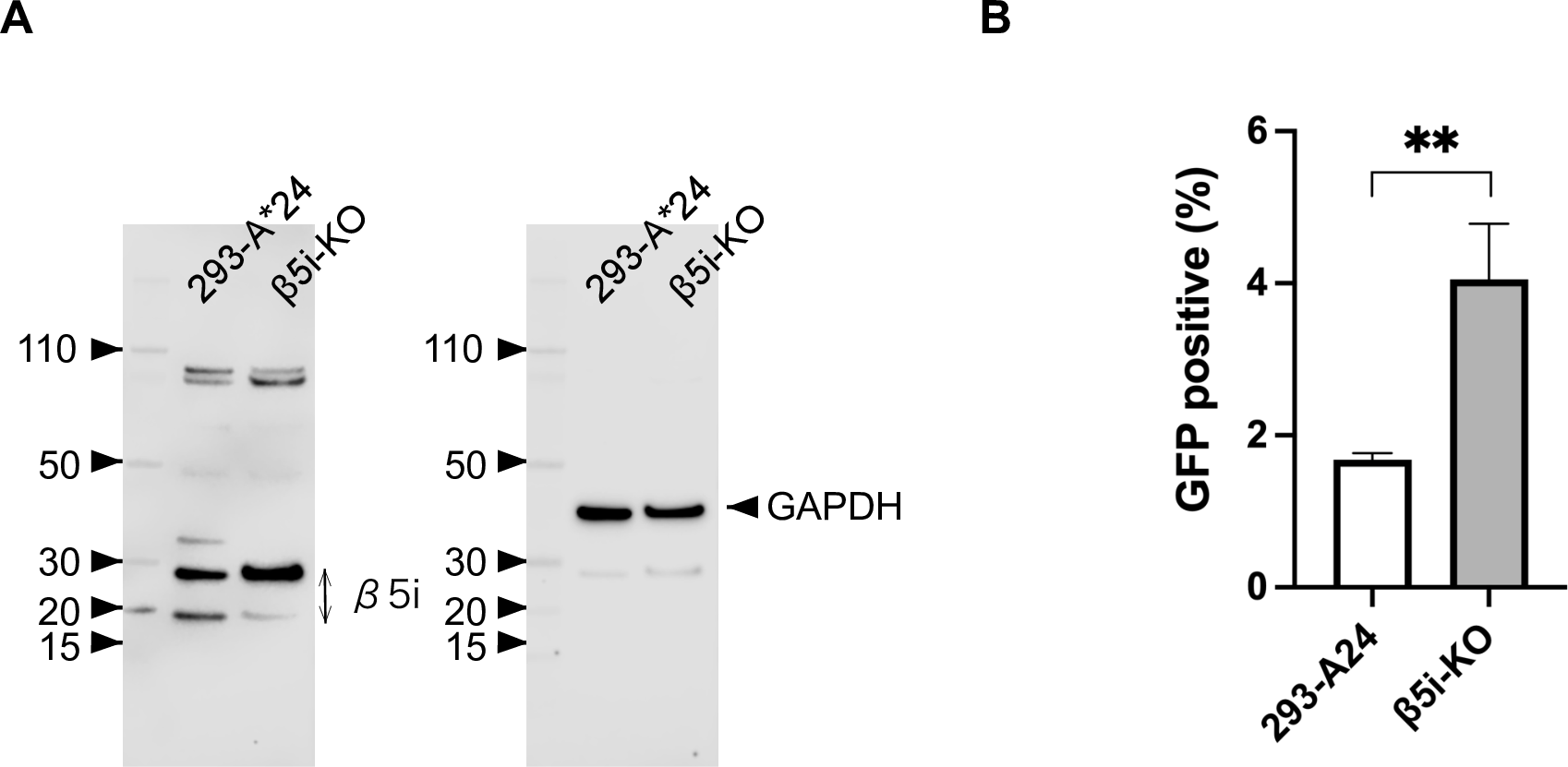

Supplement: S4 Fig — 293-A*24 cells deficient in β5i (β5i-KO) were generated in accordance with the manufacturer’s instructions (Invitrogen, #A35534, PSMB8_C1). (A) Representative western blots were used to probe β5i and GAPDH in 293-A*24 and β5i-KO cells. (B) A reduction in the level of mature β5i expression in 293-A*24 cells has been observed to enhance the activity of reporter T cells. Data are represented as the mean ± SD of three independent experiments and Unpaired t test was used. **, p<0.01. (TIF) [file pone.0308330.s004.tif]

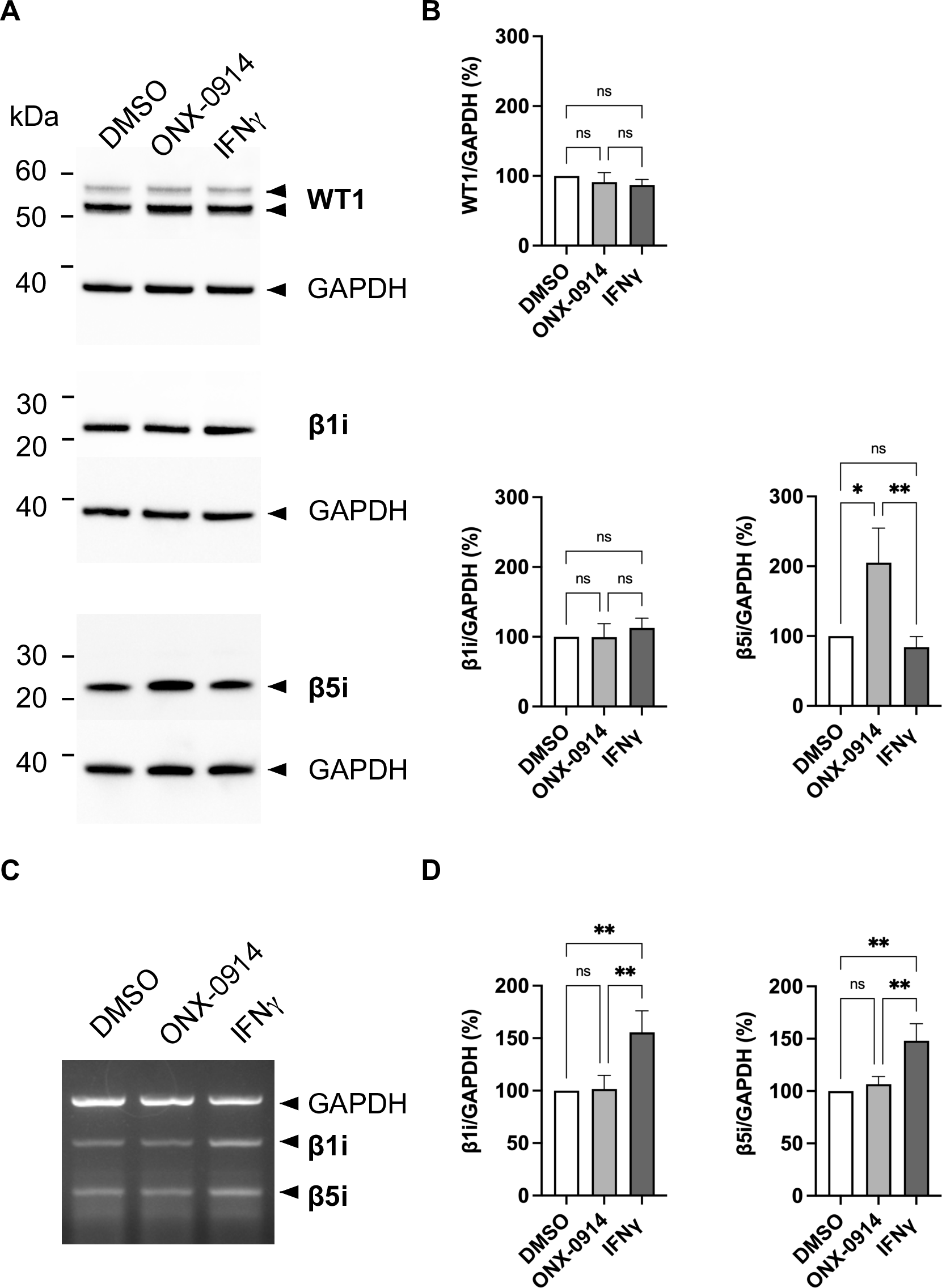

Supplement: S5 Fig — (A) Representative western blots were used to probe WT1, β1i, β5i, and GAPDH in MESO-4 treated with ONX-0914 (20 nM) and IFNγ (10 ng/ml) for 3 hours. (B) Densitometry values of expression relative to GAPDH are indicated. Increased expression of β5i protein by treatment with ONX-0914 indicates that ONX-0914 is mainly bound to the β5i protein. (C) A representative gel shows a typical RT-PCR analysis of β1i (322 bp) and β5i (172 bp) mRNAs with GAPDH (540 bp) as the internal control in MESO-4 treated with ONX-0914 (20 nM) and IFNγ (10 ng/ml) for 3 hours. RT-PCR products were analyzed on a 2.0% MetaPhor™ agarose gel. (D) The bar graph represents the results of the quantitative analysis of β1i and β5i mRNA levels normalized against GAPDH. MESO-4 treated with IFNγ had significantly upregulated β1i and β5i mRNAs but did not change with ONX-0914 treatment. Data are represented as the mean ± SD of three independent experiments and Tukey’s multiple comparisons test was used. ns, not significant; *, p<0.05; **, p<0.01. (TIF) [file pone.0308330.s005.tif]

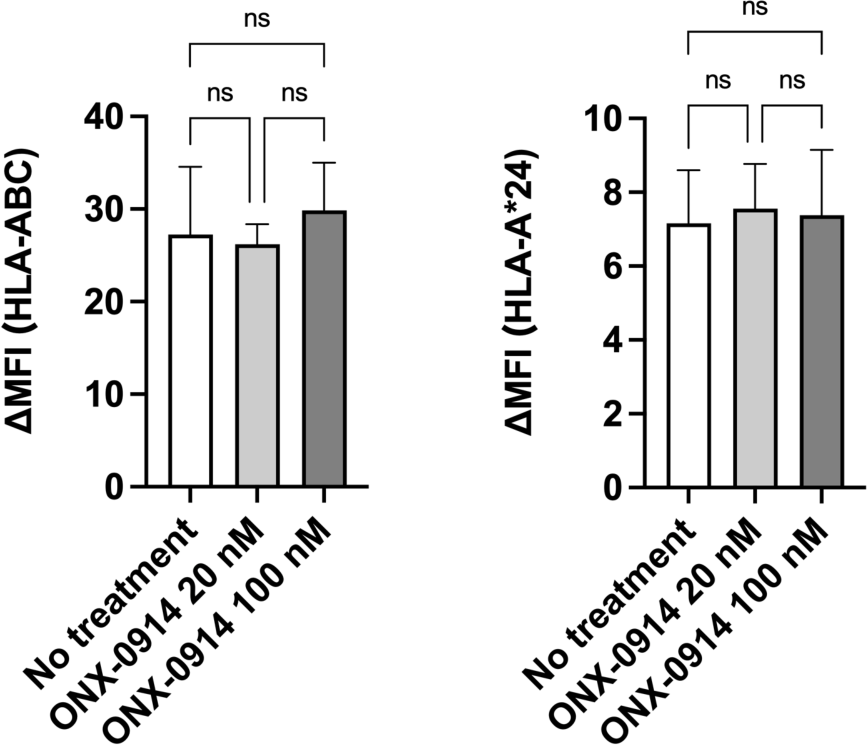

Supplement: S6 Fig — Flow cytometric analysis results showing MESO-4 cell surface expression of total MHC-I (β2-microglobulin) and HLA-A*24. ΔMFI (mean fluorescence intensity (MFI) test-MFI isotype control) is shown. Data are represented as the mean ± SD of three independent experiments and Tukey’s multiple comparisons test was used. ns, not significant. (TIF) [file pone.0308330.s006.tif]

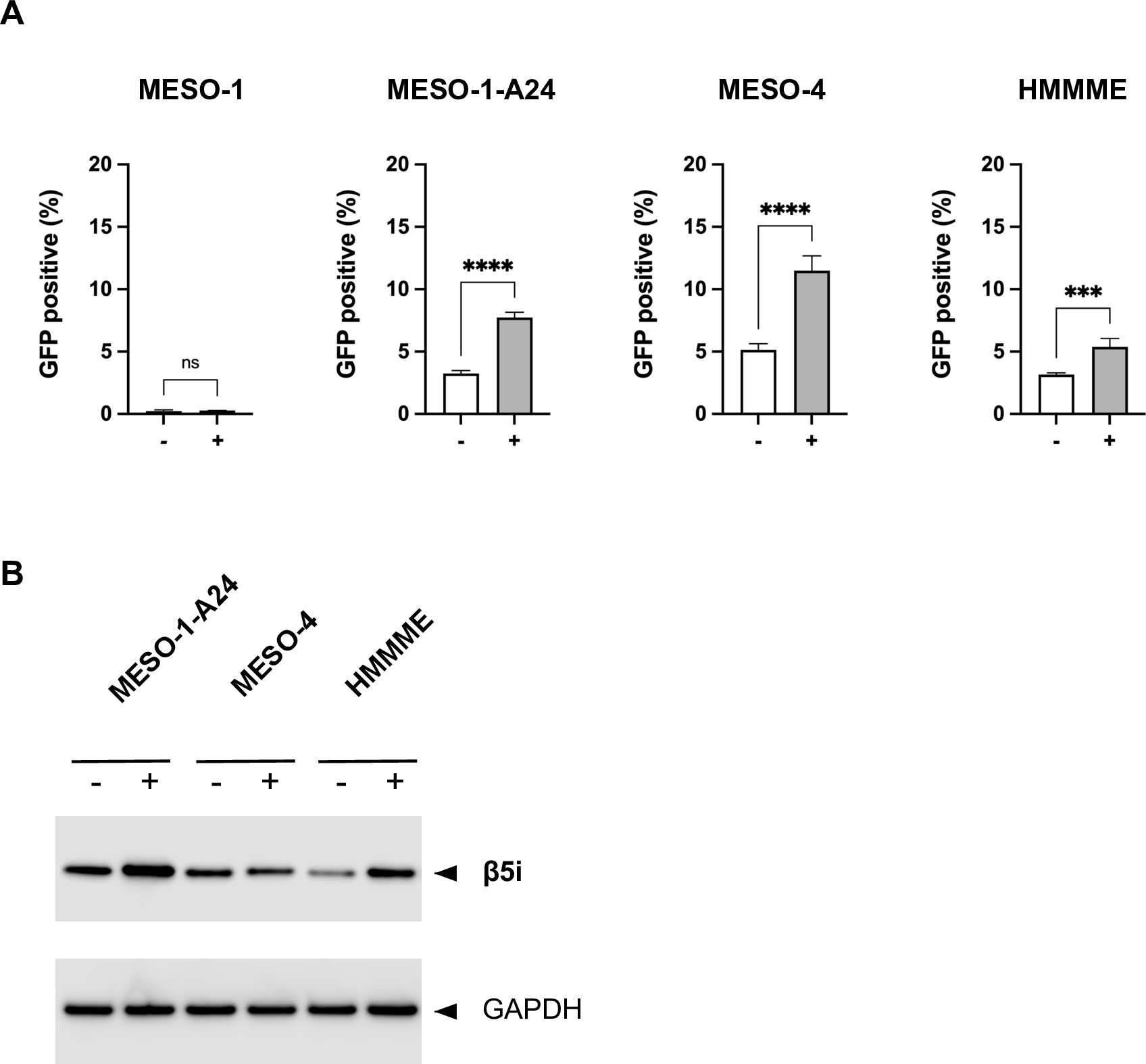

Supplement: S7 Fig — (A) MESO-1 expressing HLA*A24 are immunogenic to reporter T cells, and ONX-0914 treatment (indicated by +) further enhances their immunogenicity. Data are represented as the mean ± SD of three independent experiments and an unpaired t-test was used. (B) β5i immunoproteasome subunit expression was analyzed by western blotting. Mesothelioma cells were treated with 100 nM ONX-0914 for 3 hours (indicated by +), and the protein expression of β5i was analyzed by western blotting. Treatment with ONX-0914 resulted in a band shift of β5i, suggesting that ONX-0914 binds to β5i. (TIF) [file pone.0308330.s007.tif]

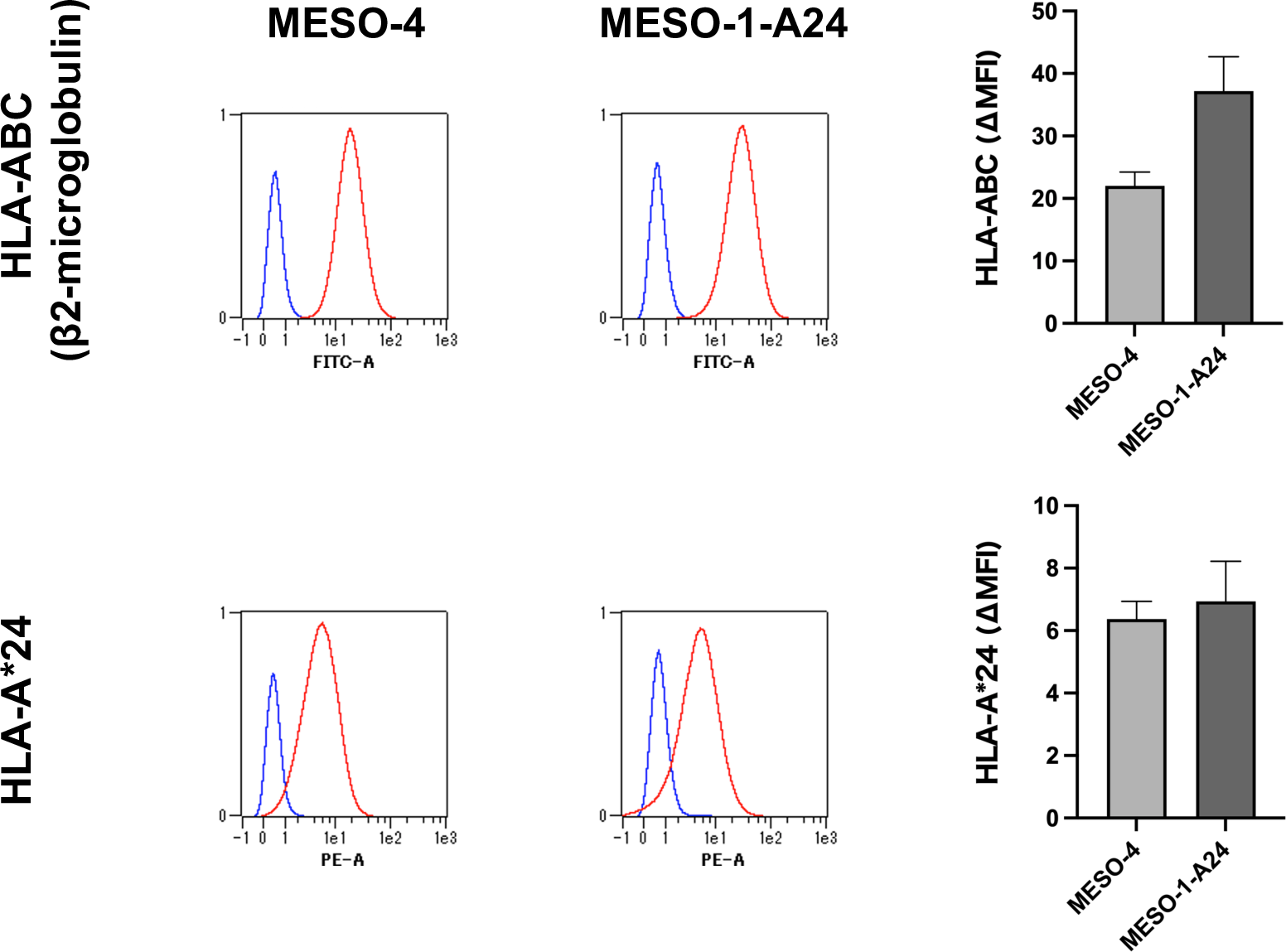

Supplement: S8 Fig — Human HLA-A*24:02:01 cDNA was provided by RIKEN BRC through the National BioResource Project of the MEXT, Japan (cat. RDB02871) (1). HLA-A*24:02:01 cDNA was excised using NheI and NotI restriction enzymes and cloned into a PB514B-2 vector (SBI System Bioscience). The cDNA vector and PB210PA-1 super PiggyBac transposase expression vector were introduced into MESO-1 using Nucleofector I (LONZA, Kit V, program T-20). HLA-A*24 expressing MESO-1 (MESO-1-A24) was selected and established in RPMI medium containing 10% FBS supplemented with 0.4 μg/ml of puromycin. The expression of HLA-A*24 was confirmed by flow cytometry using an anti-HLA-A*24 antibody (MBL, clone 22E1, # K0209-5). Reference 1. Akatsuka Y, Goldberg TA, Kondo E, Martin EG, Obata Y, Morishima Y, et al. Efficient cloning and expression of HLA class I cDNA in human B-lymphoblastoid cell lines. Tissue Antigens 2002;59(6):502–11. (TIF) [file pone.0308330.s008.tif]
