# Supplementary material for: Enhancing the immunogenicity of Wilms tumor 1 epitope in mesothelioma cells with immunoproteasome inhibitors
Source: PLoS One. 2024 Aug 8;19(8):e0308330. doi: 10.1371/journal.pone.0308330 (PMC11309442; doi:10.1371/journal.pone.0308330)

Figure 2B.

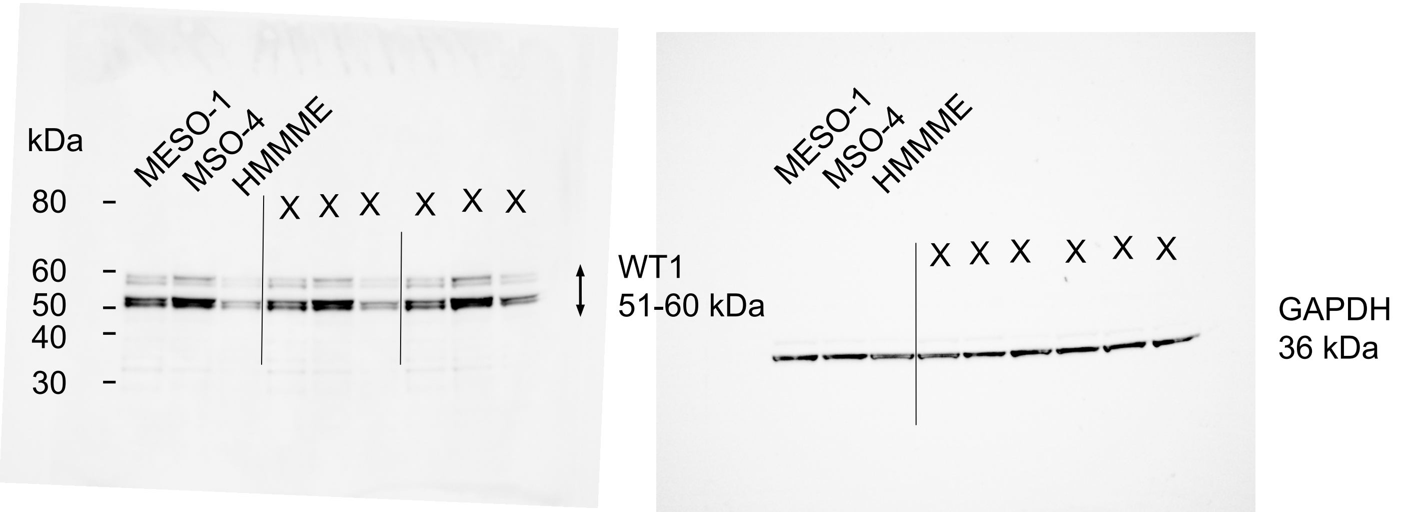
kDa
MESO-1
MSO-4
HMMME
80
60
50
40
30
X X X X X X
WT1
51-60 kDa
MESO-1
MSO-4
HMMME
X X X X X X
GAPDH
36 kDa

Figure 5A.

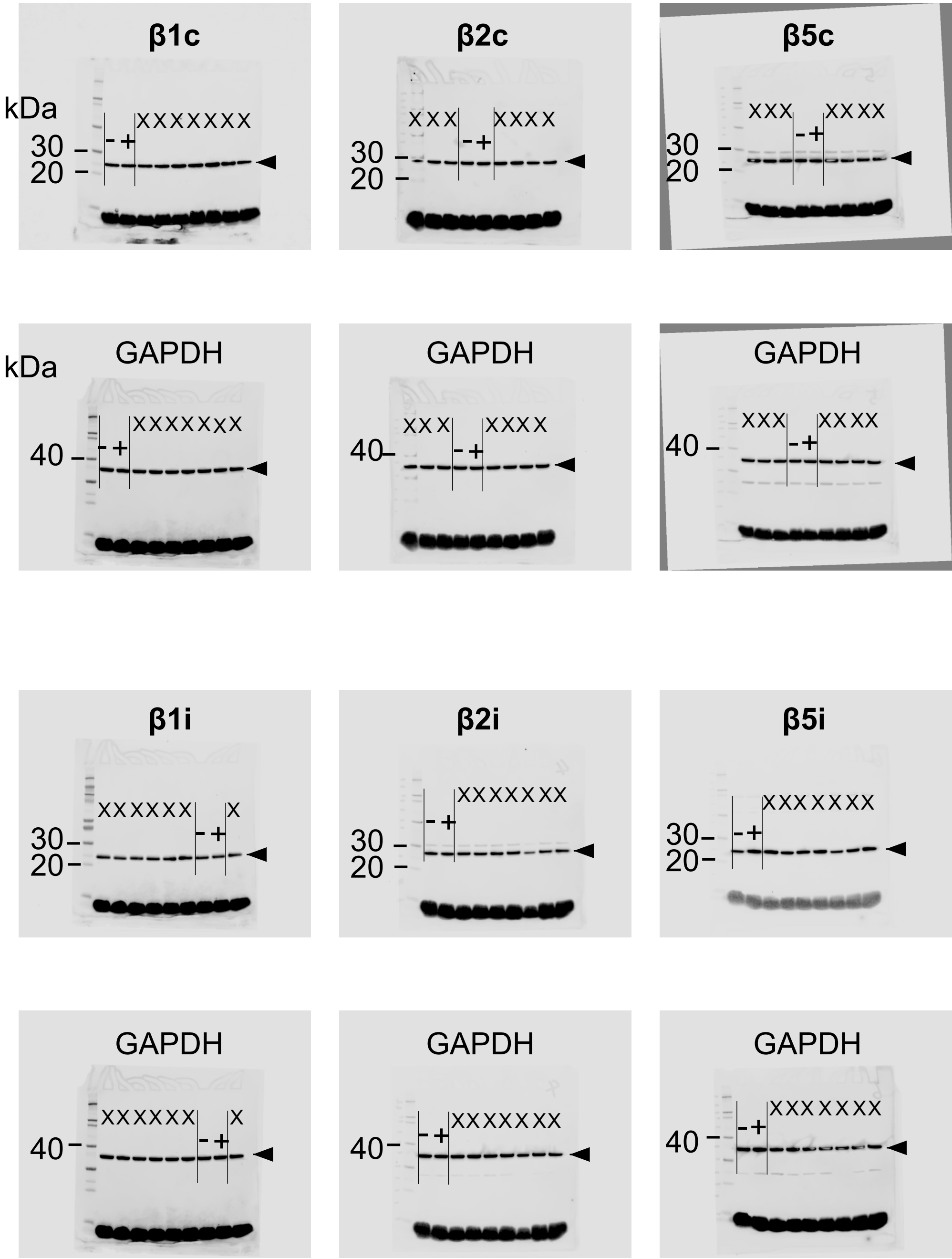
β1c
β2c
β5c
kDa
30
20
GAPDH
GAPDH
GAPDH
kDa
40
β1i
β2i
β5i
30
20
GAPDH
GAPDH
GAPDH
40

Figure S5A.

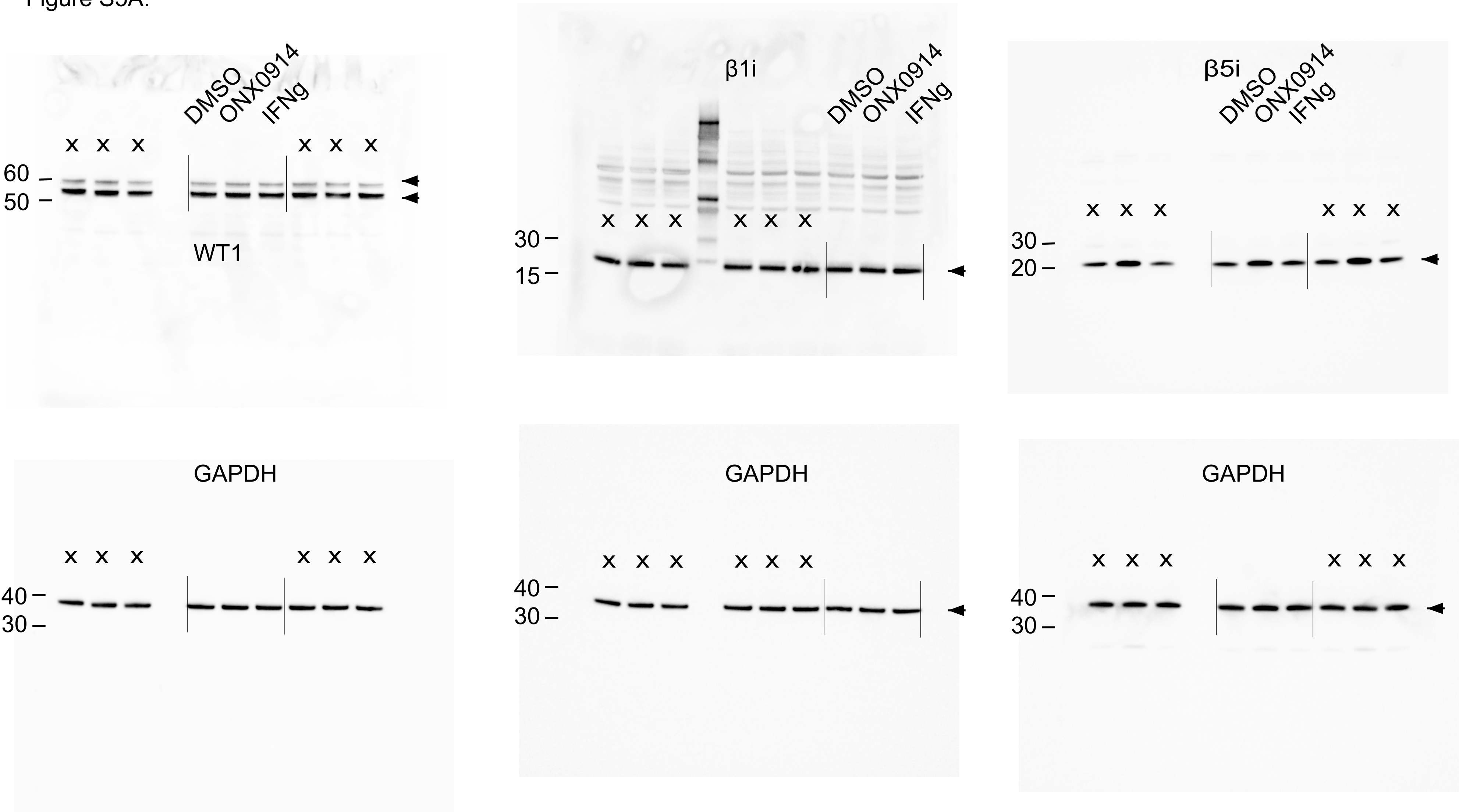

Figure S7B.

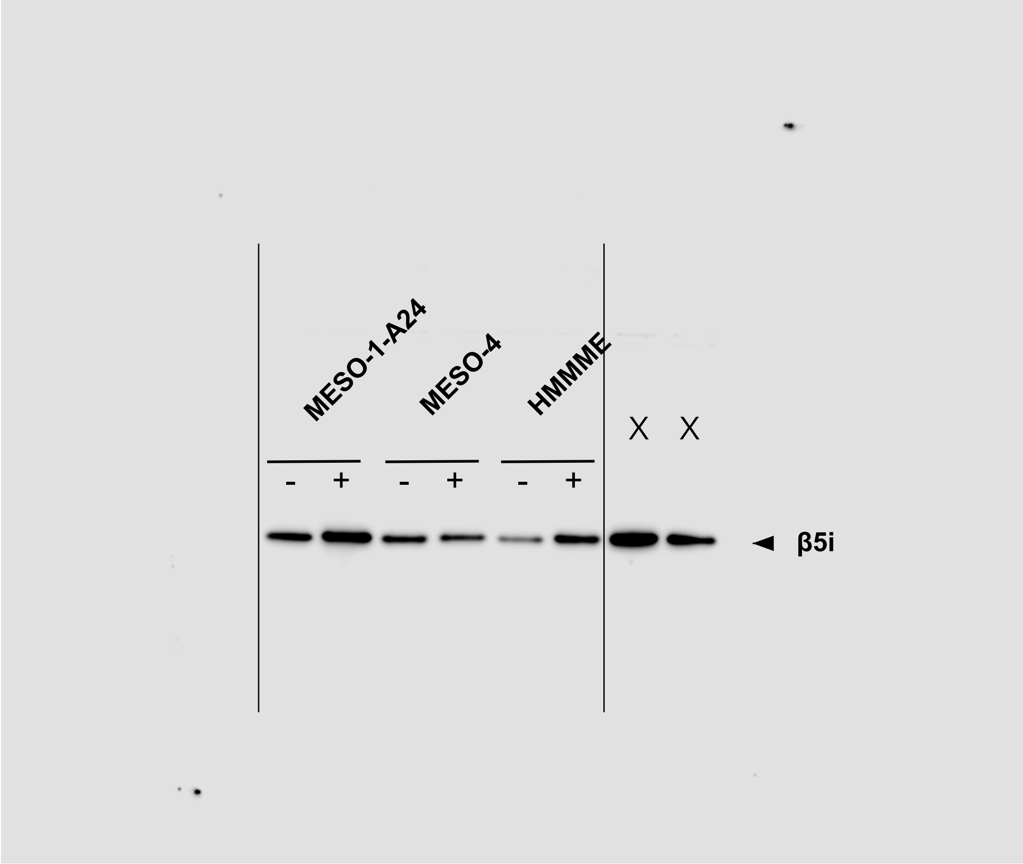

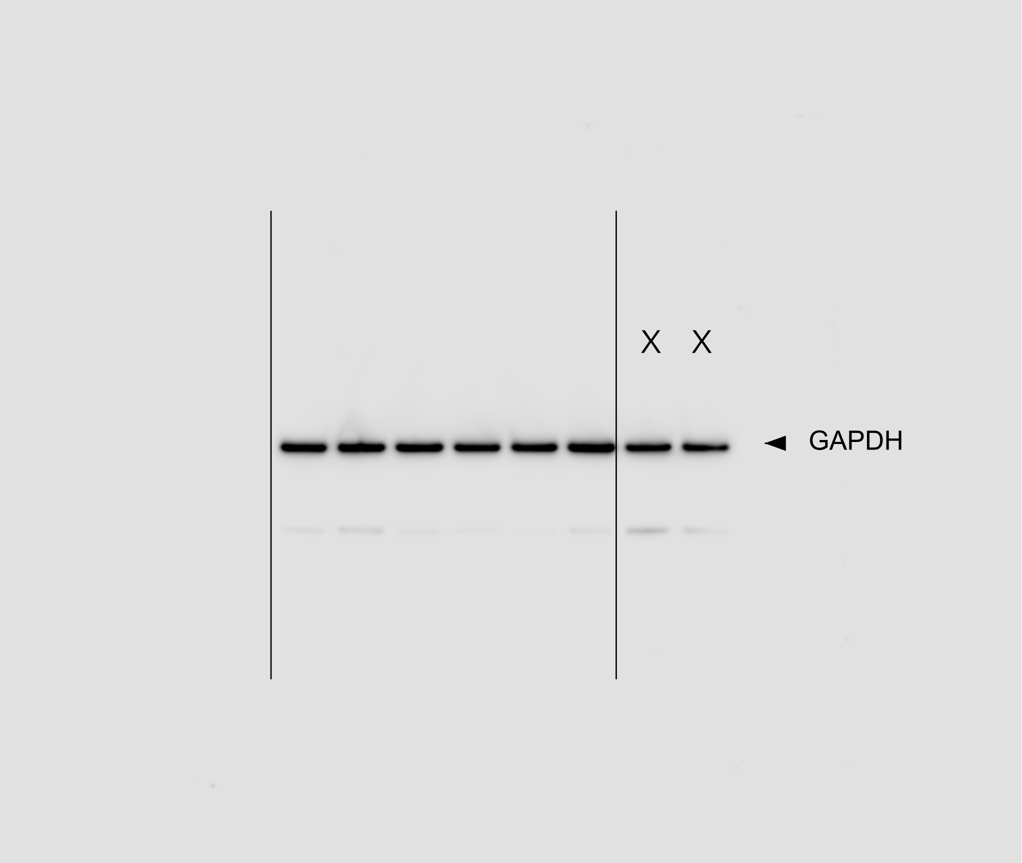

Supplement: S9 Fig — (PDF) [file pone.0308330.s009.pdf]
